# Radio-Absorbing Materials Based on Polymer Composites and Their Application to Solving the Problems of Electromagnetic Compatibility

**DOI:** 10.3390/polym14153026

**Published:** 2022-07-26

**Authors:** Alexander Fionov, Ivan Kraev, Gleb Yurkov, Vitaly Solodilov, Alexander Zhukov, Anastasia Surgay, Iren Kuznetsova, Vladimir Kolesov

**Affiliations:** 1 Kotelnikov Institute of Radio Engineering and Electronics of RAS, 125009 Moscow, Russia; asfionov@gmail.com (A.F.); ya.a1997@yandex.ru (A.S.); kuziren@yandex.ru (I.K.); 2 Institute of Biochemical Physics Named after N.M. Emanuel Russian Academy of Sciences, 119334 Moscow, Russia; nikel1311@mail.ru; 3 N.N. Semenov Federal Research Center of Chemical Physics Russian Academy of Sciences, 119991 Moscow, Russia; ygy76@mail.ru (G.Y.); vital-yo@yandex.ru (V.S.); zhukov765311@yandex.ru (A.Z.); 4 Department of Structurally Sensitive Functional Materials, Bauman Moscow State Technical University, 105005 Moscow, Russia

**Keywords:** polymer nanocomposites, nanoparticles, radio-absorbing materials and coatings, materials with controlled electro-physical characteristics, electromagnetic compatibility

## Abstract

Recently, designers of electronic equipment have paid special attention to the issue of electromagnetic compatibility (EMC) of devices with their own components and assemblies. This is due to the high sensitivity of semiconductor microcircuits to electromagnetic interference. This interference can be caused either by natural phenomena, such as lightning strikes, or by technical processes, such as transients in circuits during fast periodic or random switching. Either way, interference implies a sudden change in voltage or current in a circuit, which is undesirable, whether it propagates along a cable or is transmitted as an electromagnetic wave. The purpose of this article is to review the works devoted to the development, creation, and investigation of modern polymeric nanocomposite materials used for shielding electromagnetic radiation and their effective application for solving problems of electromagnetic compatibility. Additionally, the approach to design EMI shielding complex media with predetermined parameters based on investigation of various properties of possible components is shown. In the review, all polymer composites are classified according to the type of filler. The issues of the interaction of a polymer with conductive fillers, the influence of the concentration of fillers and their location inside the matrix, and the structure of the nanocomposite on the mechanisms of electromagnetic interaction are considered. Particular attention is paid to a new generation of nanocomposite materials with widely adjustable electrical and magnetic properties. A wide class of modern filled polymeric materials with dielectric and magneto-dielectric losses is considered. These materials make it possible to create effective absorbers of electromagnetic waves that provide a low level of reflection coefficient in the microwave range. The model mechanisms for shielding electromagnetic radiation are considered in the paper. A detailed review of the electro-physical properties of polymer nanocomposites is provided. Multilayer electrodynamic media containing combinations of layers of filled polymer composite materials with nanoparticles of different compositions and manufactured using a single technology will make it possible to create electrodynamic media and coatings with the required electro-physical characteristics of absorption, transmission, and reflection. Within the framework of the two-layer coating model, the difference in the effects of the interaction of electromagnetic radiation with conductive layers located on a dielectric and metal substrate is demonstrated. It is shown that in order to achieve optimal (maximum) values of reflection and absorption of electromagnetic radiation in the appropriate frequency range, it is necessary to fit the appropriate layer thicknesses, specific conductivity, and permittivity. Such approach allows designers to create new shielding materials that can effectively vary the shielding, absorbing, and matching characteristics of coatings over a wide frequency band. In general, it can be said that the development of innovative polymer composite materials for shielding electronic devices from electromagnetic interference and excessive electromagnetic background is still an important task. Its solution will ensure the safe and uninterrupted operation of modern digital electronics and can be used for other applications.

## 1. Introduction

The intensive development of telecommunication systems in the last 30 years has led to qualitative changes in the use of the radio frequency spectrum (RFS). During this time, the RFS load with radio emissions has increased many times over. At the same time, the air is not only filled with useful signals necessary for radio transmission but is also literally clogged with unwanted emissions from radio-electronic and electrical equipment, which create unintentional interference to receiving devices and reduce the efficiency of using RFS. At the same time, the proportion of radio emissions of artificial origin constantly increases. It is necessary to note that the RFS loading is not the same in frequency bands. In recent years, it has especially increased in the ranges of meter and decimeter waves. These ranges are widely used in telecommunication systems now [1]. Since the number of users of such systems in cities is in the millions, their operation even at relatively low transmitter powers (as a rule, not higher than 60 W for base and mobile subscriber stations and a fraction of a Watt for subscriber portable stations) led to a significant increase in RFS load. At the same time, with the development of electric and automobile transport and the widespread use of electrical and high-frequency equipment for various purposes, the RFS load in cities and industrial centers grew due to radio emissions that create industrial and contact interference. As a result, the field strength levels of only the background load component in some areas can reach 15–20 μV/m or more. At the same time, the sensitivity of modern receivers that characterizes the minimum levels of received signals converted to a receiving antenna is calculated in units and even fractions of μV/m. Under operating radio-electronic systems (RES), it is necessary to find ways to ensure the joint operation of RES with the required quality in conditions of limited frequency, time, and territorial resources. Electromagnetic compatibility of devices is its ability to simultaneously function in real operating conditions with the required quality under the effect of unintentional electromagnetic interference and not create unacceptable electromagnetic interference to other devices.

In order to prevent the unintentional release of electromagnetic radiation (EMR) generated in various electronic devices into the external space as well as to exclude the influence of radiation on individual sensitive elements inside radio engineering devices, it is necessary to solve problems of electromagnetic compatibility. It is known that metals reflect almost all electromagnetic radiation incident on them; therefore, metal screens are not used in many devices since the reflected radiation causes interference and disables radiation-sensitive devices. The radiation reflected from metal screens can be significantly reduced by using radio-absorbing materials (RAM) and coatings that attenuate EMR [2].

Electromagnetic shielding is the primary method for achieving electromagnetic compatibility (EMC) in terms of immunity to electromagnetic fields as well as compliance with the requirements for the level of radiated interference. Installing screens on noise-emitting elements ensures the separation of signals necessary for the operation of radio-electronic equipment and increases the selectivity of receivers, the noise immunity of sensitive equipment, the purity of the generator signal, and the accuracy of the devices. The correct choice of shielding method, shield material, and its design is very important at the initial design stage since it will determine the possibility of successfully passing EMC tests and the high-quality functioning of the developed equipment.

The meaning of shielding is to weaken the electric, magnetic, and electromagnetic fields in the space where these fields cause a negative effect. Screen performance depends on configuration, geometric dimensions of the screen, frequency or rate of field change, dielectric and magnetic permeability, and electrical conductivity of the screen material.

**Figure 1:**
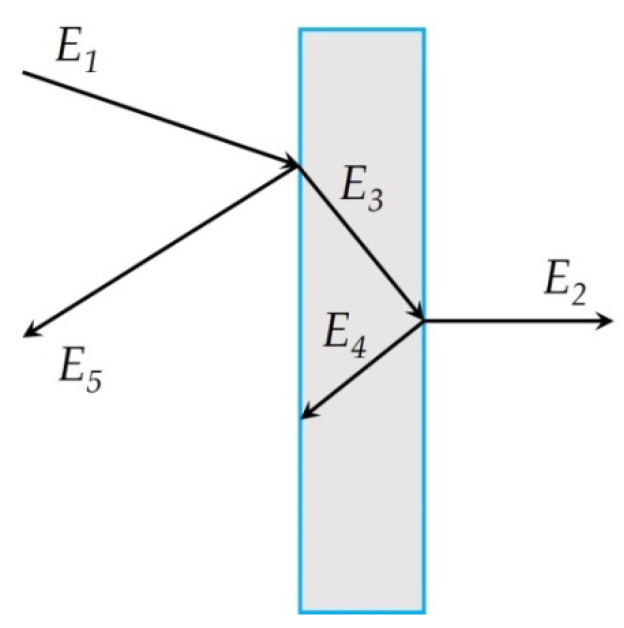
Interaction of an electromagnetic wave with a screen.

The model of interaction of an electromagnetic wave with a screen is shown in Figure 1. The main characteristic of the screen is the screening factor. An electromagnetic wave is reflected from each interface between the media, and absorption occurs in the thickness of the material.

In the general case, the screening factor *K_e_* (dB) is the ratio of the intensity of the electromagnetic field measured before the installation of a continuous endless screen and after its installation. The resulting shielding factor is the sum of the reflection and absorption losses. The shielding factor is:

*K_e_* = 20 × log(*E_1_/E_2_*), (1)

*E_2_ = E_1_ − E_3_ − E_4_ − E_5_* (2)

where *E_i_* are the intensities of the corresponding electromagnetic waves: *E_1_* is the incident wave; *E_2_* is the transmitted wave; *E_3_* is the absorbed wave; *E_4_* is the re-reflected wave; *E_5_* is the reflected wave.

For shielding permanent magnetic fields and low-frequency electromagnetic fields, where the magnetic component predominates, materials with high magnetic permeability are needed. It is known that the higher the magnetic permeability of the material, the higher the shielding factor. For shielding over a wide frequency range, multilayer materials are best suited. A promising method for creating broadband absorbing materials is the use of materials with special dispersion laws for the dielectric and magnetic permeability.

Therefore, to create an effective screen, it is necessary to fit the material, its structure, and thickness depending on the component of the electromagnetic field that needs to be screened.

Recently, fundamentally new classes of materials have appeared, such as filled polymers, conductive polymers, nanostructured polymer composites, magnetic composites, and products filled with carbon-containing materials [3]. It should be noted that the development of polymeric materials with specific properties, in particular, with special electrical and magnetic characteristics, namely antistatic, electrically conductive, radio absorbing, electret, piezo-, and pyroelectric, is currently one of the most important areas in materials science [4].

One of the important aspects of the development of electronic equipment is the issue of EMC of devices with their own components and assemblies. This is due to the high sensitivity of semiconductor microcircuits to electromagnetic interference. This interference can be caused either by natural phenomena, such as lightning strikes, or by technical processes, such as transients in circuits during fast periodic or random switching. Either way, interference implies a sudden change in voltage or current in a circuit, which is undesirable, whether it propagates along a cable or is transmitted as an electromagnetic wave.

The aim of this paper is to review the works devoted to the development, creation, and investigation of modern polymeric nanocomposite materials used for shielding electromagnetic radiation and their effective application for solving problems of electromagnetic compatibility. In the review, all polymer composites are classified according to the type of filler. The process of their manufacture is explained in detail. The issues of the interaction of a polymer with conductive fillers, the influence of the concentration of fillers and their location inside the matrix, and the structure of the nanocomposite on the mechanisms of electromagnetic interaction are considered. Particular attention is paid to a new generation of nanocomposite materials with widely adjustable electrical and magnetic properties. The model mechanisms for shielding electromagnetic radiation are considered in the paper. The review ends with consideration of some practical solutions in the development of multilayer coatings, which illustrate the possibility of obtaining almost any relationship between the coefficients of reflection, transmission, and absorption of an electromagnetic wave when interacting with such a coating. Such approach allows designers to create new effective shielding materials that can effectively vary the shielding, absorbing, and matching characteristics of coatings over a wide frequency band.

## 2. Radio-Absorbing Materials and Technologies for Their Production

Radio waves cover a fairly wide spectrum of the frequency range (the range of waves used is from ultra-long to millimeter; the range is from centimeters and meters to thousands of kilometers) and are harmonic signals modulated in amplitude and frequency. The main source of powerful electromagnetic radiation is an antenna that radiates a flow of electromagnetic energy in a directionally or non-directionally into the surrounding space [5].

RAMs are widely used in special-purpose equipment. These materials are designed to ensure the electromagnetic compatibility of radio-electronic equipment and antenna systems [6,7]. The main radio engineering parameters that characterize the RAM are: the values of the real and imaginary parts of the dielectric and magnetic permeability as well as the reflection coefficients (*R*) and/or transmission coefficients (*T*) in the frequency ranges of the microwave spectrum.

It is known that an ideal single-layer absorber of electromagnetic waves (EMW) is a material with equal dielectric and magnetic permeability. In this case, the reflections from the front boundary of the material will tend to zero [8].

### 2.1. RAM Classification

In real conditions, obtaining RAM with the same values of dielectric and magnetic permeability in the frequency range is an extremely difficult task due to differences in the physical processes that determine these parameters.

RAM can be classified according to various criteria, for example, according to the operating range of effective action: (i) broadband-, when the *R* values do not exceed a given value in the frequency range *λmax*/*λmin* ≥ 10; (ii) narrow-range-; (iii) tunable-; and (iv) selective-type RAM.

According to the structure of RAM, they are divided into (i) single-layer constant composition, (ii) gradient-type (multilayer materials with a stepwise change in electrodynamic characteristics from the front boundary to the rear), (iii) interference-type, (iv) geometrically inhomogeneous (for example, spike-like materials), and (v) combined, i.e., materials with a possible combination of gradient and interference structures.

Most of the existing interference-type RAMs depending on their structure can be divided into the following groups: (i) Salisbury screen based on an outer layer of a thin-layer conductive film located on a dielectric layer screened from the back side, (ii) Dallenbach screen based on absorbing layers placed on a conductive shielding substrate, and (iii) Jaumann screen based on multilayer alternating structures of dielectric and conductive layers [9,10]. As a rule, gradient-type RAMs have more broadband EMR absorption compared to similar single-layer absorbers [11]. A special case of such RAMs are materials with an unfilled input layer with respect to external electromagnetic effects (“soft input”). The values of dielectric permittivity *ε* of such a layer are as close as possible to *ε* of the external environment at magnetic permeability *μ* = 1. The subsequent layer or layers are distinguished by higher values of *ε* and *μ* that increase as one approach to the reflective substrate [12]. The thickness of the outer input layer is usually greater than the thicknesses of subsequent layers.

RAMs can be divided into materials of (i) magnetic-type, (ii) non-magnetic-type, and (iii) combined materials depending on the functional filler used. Materials of the magnetic type interact with the H-component of the electromagnetic field and have magnetic losses. Non-magnetic materials interact with the E-component of the electromagnetic field and have only dielectric losses. Combined-type materials contain both magnetic and non-magnetic conductive fillers.

RAMs classification can be carried out according to a number of other features technological, matrix composition, etc. Currently, smart coatings, including active (controlled) radio-absorbing structures, are becoming increasingly important [13]. The principle of operation of such structures is based on the use of external sensors that capture the effective EMR on the object and controlled layers designed to process and restructure the parameters of incoming signals in order to reduce them. In this connection, a new classification of RAM appears: (i) active and (ii) passive. Active RAMs are able to provide values of *R* of EMR from the boundary of the medium and free space, which is close to zero at any polarizations and angles of incidence [14]. Any elements capable of changing the electrodynamic parameters of the material, for example, frequency selective gratings with pin diodes, can be used as active structures for such RAMs and radio-absorbing coatings (RAC). The main preference is given to pin diodes due to their low weight and ease of control by changing the values of the applied external voltage [15].

At present, active RAMs are already used in the fifth-generation Japanese fighter X-2 [16]. Active RAMs and RACs are prone to self-excitation, i.e., generation of their own EMR, which is an unmasking factor. To match the intelligent sensors of active RAMs and RACs, in some cases, it is necessary to use passive radio engineering materials. These materials could be composites with functional fillers. In most cases, obtaining passive RAMs is characterized by ease of manufacture and lower economic costs compared to active structures. In this connection, the works aimed at creating and improving traditional (passive) RAMs do not lose their relevance.

The vast majority of traditional passive RAMs and RACs are composites based on matrices with low dielectric constants and functional fillers. Ceramic [17], polymeric [18], textile [19], mineral materials [20], etc., can be used as matrices.

### 2.2. RAM’s Matrixes and Fillers

The choice of matrix and filler is determined by the purpose and operating conditions of the RAM. The main disadvantages of RAMs based on magnetic fillers are high values of bulk density as well as a low operating temperature range. One of the common advantages of such materials is to achieve lower values of *R* of EMR in thinner layers compared to non-magnetic type materials as well as to reduce the contribution to backscattering associated with edge diffraction and surface waves.

In this connection, in most cases, magnetic-type composites are used as coatings. Ferrites and powders of ferromagnetic metals are among the most characteristic magnetic fillers used in the creation of RAMs [21]. To ensure a wider operating temperature range of RPM, as a rule, non-magnetic type fillers are used, which are various electrically conductive functional particles: carbon black, graphite, carbon-containing fibers, powders of diamagnetic metals and their compounds, carbon nanotubes (CNT), graphene, etc. [22]. The microwave and electrophysical properties of RAM depend on the composition, thickness, and number of layers as well as on the concentration of functional fillers. By varying these parameters, it is possible to give composite materials (CM) the necessary radio characteristics.

Soot-filled RAMs possess low values of bulk density; however, to achieve low values of *R* in the ranges of the microwave spectrum, thicknesses of more than 10 mm are required [23]. There are a large number of RAMs made using carbon fiber fillers. It is known that such fillers can be used both in materials for anechoic chambers [24] and for structural reinforced plastics [25,26].

### 2.3. RAM with Magnetic Fillers

Of greater interest are RAMs with high values of high-frequency dynamic magnetic permeability [27]. Such materials include CMs based on powders of ferromagnetic metals and hexagonal ferrites [28]. There is a known RAM based on nickel-zinc ferrite [29]. It provides efficient EMR absorption in the frequency range from 30 MHz to 1000 MHz. RAMs based on ferromagnetic metals; for example, metals of the iron triad (Fe, Ni, Co) as well as their various alloys and compounds have become widespread [30]. Carbonyl iron powders are of great interest for radio engineering applications and research [31]. For the antennas, developing the RACs based on an epoxy elastomer and carbonyl iron powder is used [32].

The shape and size of the functional filler has a significant impact on the radio-absorbing properties of the RAM [33]. A classic example of varying the electrodynamic properties of RAM based on carbon-containing fibers is a change not only in the concentration of the filler but also in its linear dimensions, for example, by cutting the fiber into lengths from tenths of mm to tens of mm [34].

It is known that powders of ferromagnetic metals and their compounds in the form of flakes and plates significantly increase the values of the dielectric and magnetic permeability of composites based on them compared with materials containing similar spherical ferromagnetic particles [35,36]. This is explained by the higher values of the average polarizability of composites with lamellar inclusions due to the small values of the form factors in the particle planes compared with composites containing spherical inclusions. As a rule, plates and flakes of ferromagnetic powders are produced by high-energy grinding in a closed-circuit bead mill in various organic media, for example, in ethanol and heptane [37]. Commercially available spherical ferromagnetic powders are usually used as the initial raw material for obtaining lamellar particles [38].

The disadvantage of RAM based on lamellar particles at their concentration above 40–50 vol. % is an increase in the values of the dielectric constant (more than a hundred).

The magnetic characteristics of powders of the *La_x_Nd_2−x_Fe_17_* (*x* = 0.0, 0.2, 0.4, 0.6) alloy of various morphologies and the electrodynamic properties of corresponding RAMs were studied. The shape changing of the powders was carried out by high-energy grinding. The obtained powders of the investigated flake-shaped alloys provided the *R* of EMR for RAM samples less than −10 dB in a wide frequency range at a thickness of 2 mm. It has been found that the minimum values of the *R* of EMR amounted to −32.5 dB at 9.8 GHz [39].

Among the known magnetic materials for microwave applications, a special place is occupied by ferromagnetic films and CMs (laminates). CMs (laminates) based on ferromagnetic and polymeric films have high magnetic losses in the LF region and have a bulk density not exceeding 2–3 g/cm^3^.

As a rule, obtaining a thin layer of a ferromagnetic metal or other material, such as hydrogenated carbon, is achieved using methods of ion-plasma magnetron sputtering on a substrate. The substrates can be polymer films, woven, non-woven materials, etc. The granular films of hydrogenated carbon with nanoparticles of ferromagnetic metals (*Co*, *Ni*) and corresponding RACs were developed [40]. The value of *R* of EMR for obtained RAMs did not exceed −10 dB in the frequency range from 7 to 70 GHz. RAMs were multilayer structures based on aramid fabric coated with films of hydrogenated carbon with different concentrations of ferromagnetic metals from layer to layer and, consequently, varying values of dielectric and magnetic permeability [40]. The films were deposited on the fabric surface by the method of ion-plasma magnetron sputtering. The RAM based on thin films of amorphous hydrogenated carbon with ferromagnetic nanoparticles deposited on a flexible substrate of aramid fabric by ion-plasma magnetron sputtering was developed [41]. According to the results obtained, such RAMs provide a high level of EMR absorption with *R* is in the range of −10 to −30 dB in the microwave frequency range. These materials are characterized by ultra-wideband.

A material that consists of two layers of polymer nanofibers bonded with a radio-transparent material is presented in [42]. A film of hydrogenated carbon with embedded ferromagnetic or ferrimagnetic particles is deposited on each layer of polymer nanofibers by vacuum sputtering. This material provides effective EMW absorption in the frequency range from 5 to 70 GHz at small thicknesses (no more than 2 mm). However, in real conditions, the application of such materials to the surface of a number of objects is an extremely difficult task due to low manufacturability and the need to create additional protective coatings and materials that can improve the physical, mechanical, and tribological properties, for example, erosion resistance. The RAMs described are also characterized by high cost. RAMs based on magnetic fibers have attracted the attention of many researchers. Micro wires can be referred to such magnetic materials [43]. The cores of micro wires are characterized by high values of magnetic permeability. The high value of the dynamic magnetic permeability of fibrous ferromagnetic materials is manifested in the direction along the axis of their fibers; however, the disadvantage is the high dielectric response, which exceeds the magnetic one.

Recently, RAMs based on nanosized fillers and powders with a nanosized crystalline structure have become increasingly important [44,45]. It is known that crystalline ferromagnetic powders have a large domain size, and therefore, the process of rotation of the magnetization vectors is hindered [46]. Such shortcomings are absent in nanocrystalline soft magnetic alloys with a set of *α-Fe* or *α-(Fe,Si)* nanocrystals in the superparamagnetic state and located in the residual amorphous matrix [47].

### 2.4. RAM with Nanocarbon Fillers

Nanosized electrically conductive fillers, for example, fullerenes, graphene [48], and CNTs [49], have a wide prospect for creating appropriate RAMs due to their small size and high electrical conductivity. The presence of thin fibrous inclusions in the composition of the CM makes a more significant contribution to the dielectric losses. Inclusions with large linear dimensions limit the technical applications, such RAMs. For example, sheet materials based on carbon fibers of millimeter size are characterized by anisotropy of the permittivity along and across the sheet plane. The clusters can appear in CNT-based RAMs. Its presence as well as their shape and geometric sizes, the value of their capacitances, and conductivities are affected by the frequency dispersion of the permittivity of the material and the expansion of the frequency range. It should be noted that the electrical resistance of contacts between particles and clusters can be much higher than the ohmic resistances inside the particles themselves. In some cases, this effect can make the main contribution to the electrodynamic properties of the material [50,51].

It is known RAMs based on *BaFe_12_O_19_* (BHF) /MWCNTs/PANi nanocomposite [52]. As it was observed due to the presence of PANi, the absorption increased extraordinarily. The absorber exhibited a maximum reflection loss of −24.2 dB at 11.6 GHz.

The manufacturing method and properties of structural RAM based a polyurethane composition with CNTs deposited on walls of honeycomb plastics are described in [53]. By varying the content of CNTs in the compositions, the possibility of tuning the radio technical characteristics of the RAM was shown. The best characteristics on a single-layer composite were obtained at a CNT concentration of 5.6 wt. % (the minimum EC value of *R* was −24 dB at the resonant frequency of the operating frequency range of 2–18 GHz). With an increase in the number of layers of the functionalized honeycomb, a further decrease in the values of *R* was noted.

The methods for regulating the electromagnetic parameters of RAMs based on polyurethane with multiwalled CNTs introduced into its volume are described in [54]. The characteristics of both CNTs and composites were changed by modifying the CNT surface with ferromagnetic metal oxides of various concentrations.

In a number of cases, in order to achieve lower values of the *R* of EMR and expand the operating RPM range, groups of various functional fillers are used. These fillers are introduced as a mixture into the volume of matrices. Both mixtures of magnetic powders and non-magnetic particles in combination with magnetic inclusions can be used as such fillers [55,56,57].

It is known multilayered RAMs with varying values of parts of the magnetic and dielectric permittivity from layer to layer are made on the basis of ferrite and carbonyl iron powders [58,59,60]. Another example of the technical implementation of RAM based on groups of functional fillers is the material described in [61,62]. Here, to ensure the absorption of EMR in the sub-bands of the range from 2 to 60 GHz, powders of hexagonal barium and strontium ferrites were introduced in combination with ultrafine powders of spinel ferrites and iron carbide.

A material based on latexes with functional fillers as combinations of fullerenes with powders of carbonyl iron and ferrites is described in [63]. The resulting composite ensures efficient absorption of the incident EMR energy in the frequency range from 2 to 20 GHz. The aim to broadening the operating frequency range of the RAM is solved through the use of groups and mixtures of various functional fillers. It was shown that at CNTs combining with nanosized magnesium-zinc ferrite powders (*Mn_1−x_Zn_x_Fe_2_O_4_* (*x* = 0.0 и 1.0)) the value of the *R* of EMR obtained in the frequency range from 8 to 12 GHz did not exceed −10 dB [64]. The RAMs based on CNTs and magnetite nanoparticles, providing the values of the *R* of EMR of no more than −15 dB in the frequency range from 10.2 to 18 GHz and with a thickness of no more than 3.0 mm, is described in [65].

The RAMs based on nanostructured graphene oxide and magnetite powder with various concentrations are presented in [66]. It was found that the joint introduction of the investigated functional fillers allow to control the values of the dielectric and magnetic permeability in the microwave range. For samples containing a mixture of magnetite powder and graphene oxide (at a concentration of up to 3 wt. %), lower values of the *R* of EMR in the frequency range from 2 to 18 GHz are provided compared with RAM samples based on magnetite powder alone. With a sample thickness of 1.7 mm, the values of the *R* of EMR were no more than −10 dB in the studied frequency region.

It is necessary to note that the choice of matrix material during RAM design is very important due to the need to ensure the required performance depending on the application. It is also necessary to take into account manufacturability and economic indicators.

Currently, there are promising works aimed at creating structural RAMs for various purposes [67,68]. Reinforced composites are known, which are fiberglass based on an epoxy binder containing resistive carbon fibers [69]. Another example of the implementation of a structural RAM is a laminated composite consisting of layers of fiberglass in combination with a lamellar porous structure [70]. Modern methods for modeling and obtaining structural RAMs, including those with a gradient structure, are 3D-printing technologies [8,71]. It should be noted that materials with a large number of air-filled cavities and cells in its volume have lower values of dielectric permittivity compared to similar close-packed matrices. Classical examples are porous-cellular (foam plastics [72,73], spheroplastics [74]), and porous-fibrous (non-woven fabrics [75,76] and mats [77]) materials.

A lightweight covering RAM based on polyacrylonitrile fibers and nickel with cobalt materials introduced into its volume and applied to its surface at the manufacturing stage is described in [78]. According to the authors’ results, the described RPM provides values of the *R* of EMR that do not exceed −20 dB in the operating frequency range.

### 2.5. RAM Based on Elastomers

A method for synthesizing a functionalized nickel–carbon porous material where the formation of *Ni* and carbon nanoparticles occurred simultaneously at the material formation stage is presented in [79]. The density of the obtained RAM was 0.1 g/cm^3^, the value of the *R* of EMR at a thickness of 2 mm at a frequency of 4.5 GHz was no more than −10 dB, and at a frequency of 13.3 GHz, it was −45 dB.

Increasing the operating temperature range of CM greatly expands the possibilities of their application. Organosilicon materials are among the most heat-resistant (workable up to 300–400 °C) polymer binders and compositions, including those resistant to UV radiation and water. These elastomers are widely used in the creation of RAMs with such fillers as iron nanoparticles, pyrite ash, and carbonil iron particles [80,81,82].

Taking into account the wide range of RAMs and functional fillers of various types, there are a large number of methods for their production and technologies for their manufacture. Questions on the development of promising technologies for the manufacture of RAMs with improved properties still do not lose their relevance.

The following most common manufacturing methods depending on the matrices used in composites can be distinguished. Sintering technologies of raw materials at various temperatures and holding times are usually used to create RAMs based on ceramic matrices [83,84]. For RAMs based on textile and fibrous structures, impregnation methods and needle-punched fabrication methods are used [85,86].

There are a large number ways to produce the RAMs based on polymer matrices. An important role in the choice of processing technology is played the chemical composition, rheology, thermal properties of the binder, the type of curing used, and the composition and concentration of the filling. For RAM based on thermoplastic matrices, the following processing methods are widely used: (i) molding under pressure in a press or autoclave [87], (ii) extrusion methods [88], and (iii) additive technologies [89,90]. Such methods as pouring compositions [91], pressing [92,93] and autoclave molding [94], paint and varnish application methods [95,96], technologies for manufacturing porous structures [97], and impregnation technologies [98,99] are used for production the RAMs based on fiber-reinforced polymer composites. In some cases, to expand the operating radio frequency range and ensure a set of requirements for performance characteristics (physical, mechanical, thermal, resistance to external factors, etc.), RAMs based on combinations of different materials manufactured according to their technology are used [100].

## 3. Composite Materials Based on Filled Polymers

The term “composite materials” or “composites” appeared when a most capacious name was required for a new class of materials consisting of a reinforcing component and a binder. Composite materials were also called reinforced or filled with plastics (plastic masses). Currently, the term “filled polymers” is widely used to refer to heterophase composite materials with a continuous polymer phase (matrix). In such matrices, solid, liquid, or gaseous fillers are distributed randomly or in a certain order. These substances fill part of the volume of the matrix while either reducing the consumption of scarce or expensive raw materials, or the composition is modified, obtaining the desired qualities corresponding to the purpose, technological features of production and processing, as well as the operating conditions of the products. Filled polymers are the vast majority of plastics, rubbers, paints and varnishes, polymer compounds, adhesives, and other polymer composite materials [101]. Depending on the type of polymer matrix, filled thermoplastics, thermoplastics, and rubbers are distinguished. The most common binders are: polyesters, phenols, epoxy compounds, silicones, alkyds, melamines, polyamides, polyimides, fluorocarbons, polycarbonate, acrylics, acetals, polypropylene, acrylonitrile butadiene styrene copolymer (ABS), polyethylene, and polystyrene. Fillers can be incorporated into plastics to modify the properties of thermoplastics or thermosets. The modern industry of composite materials widely varies different combinations of reinforcing components and binders. The choice of the appropriate components is determined both by technical parameters and by price. Most of the properties of the filled polymers turn out to be higher than the properties of the original components. A material whose structural purpose is the same as one of its components may also be referred to as a composite. Such materials are, for example, products covered with a polyvinyl film used in aircraft, laminated metal-plastic facings, etc. [102]. The range and applications of the filled polymers are constantly expanding.

The composites are divided into particulate-filled plastics that based on dispersed particles of various shapes, including crushed fiber, reinforced plastics that consist of reinforcing filler with a continuous fibrous structure, gas-filled plastics, and oil-filled rubbers. This dividing is independent on the filler type.

As the fillers’ various fibers, powders, microspheres, crystals and whiskers from organic, inorganic, metallic materials, or ceramics can be used. Most often, solid fillers are used to obtain filled polymers finely dispersed with particles of granular (carbon black, wood flour, *SiO_2_*, chalk, etc.) or lamellar (talc, mica, graphite, kaolin, etc.) forms as well as various fibrous materials in the form of threads, strands, bundles, canvases, mats, fabrics, paper, and nets.

Besides using dispersed fillers and chopped fiber, the main production method is mechanical mixing of the filler with a melt or solution of a polymer, prepolymer, oligomer, or monomer. For this purpose, mixers of various designs and rollers are used. Continuous fibrous preforms are impregnated with a polymeric binder. The various methods of modifying the surface of the fillers as well as the method of polymerization on fillers are used to improve the impregnation of fibrous fillers with a binder, to increase the degree of dispersion of filler particles in the matrix, and to increase the strength of the adhesive contact at the filler–matrix interface. Gas-filled materials are obtained by foaming with the help of special agents (porogens) or by mechanical foaming of liquid compositions, such as latexes. The foamy structure of the polymeric material is fixed by cooling the composition below the glass transition temperature of the polymer, curing, or vulcanization. Liquid fillers are mechanically emulsified in a binder. The subsequent transformation of it into a filled polymer matrix occurs without destroying the original emulsion structure.

The properties of composite materials are determined by the properties of the polymer matrix and filler, their ratio, the nature of the filler distribution in the matrix, and the nature of the interaction at the polymer–filler interface. By improving any characteristic of the composition, the filler can simultaneously worsen its other properties. For example, most types of carbon black increase not only strength but also the elastic modulus (hardness) of rubber and the latter is undesirable in many cases. Therefore, in each specific case, during selection of the type, concentration, and method of surface modification of the filler, it is necessary to carefully balance the effects caused by the presence of the filler and matrix in the composite.

Composite materials based on filled polymers are widely used for the development of radio engineering materials. The composites are based on dielectric carbon-chain polymers obtained by polymerization (polystyrene, polyethylene, polyvinyl chloride, polytetrafluoroethylene) or polycondensation (resole, novolac and epoxy resins, lavsan, polyimides) methods. The most popular polymer-based composites used in electrical and radio engineering are layered plastics, gas-filled plastics, and magneto-dielectrics [49,103,104,105,106,107,108,109,110,111,112,113].

Various methods can be used to synthesize composites based on filled polymers. It could be mechanical mixing of components with subsequent processing into finished products, treatment of polymer films with metal vapors, chemical reactions of metal salts in polymer solutions with subsequent isolation of the corresponding polymer, polymerization of metal-containing monomer systems, etc. [114,115,116,117,118,119].

The development of nanotechnology opens up new approaches to the creation of radio engineering materials, including materials for ensuring electromagnetic compatibility. The task of nanotechnology is to develop economically and environmentally efficient technologies for obtaining new practically important nanostructured materials and highly dispersed systems, films and coatings, functional nanostructures, and elements of nanoelectronic devices that are promising for practical applications in various fields from information and telecommunication systems, nanomechanics, nanoelectronics, optoelectronics, and catalytic systems to bionanotechnology and nanomedicine.

The creation of nanomaterials is the most promising for a number of reasons. First of all, this is due to the recently opened possibilities of an infinite variety of sizes, shapes, compositions, and structures of nanoparticles that affect the unique physicochemical properties of nanocomposites obtained by “chemical” (solution) methods. This, in turn, makes it possible to predetermine and vary the physical properties of nanoparticles before using them as “building blocks” to create nanomaterials.

Particles with sizes less than 10 nm fundamentally differ in their physicochemical properties from macro-objects since the surface/volume ratio sharply increases with decreasing particle size. In highly dispersed and highly porous materials, the surface area increases significantly, and consequently, the chemical reactivity of such objects increases, too. In addition, in bulk materials, a decrease in the size of crystallites leads to an increase its mechanical characteristics. More important is that quantum mechanical effects begin to appear in the behavior of nano-objects. This is attributed with the fact that electrons cannot be in continuous energy bands in nanoparticles, but they occupy only a few narrow energy levels, the structure of which is determined by the particle size. Such a limitation drastically changes the mechanism of the electrical conductivity of nanomaterials since the conductivity becomes a quantized quantity; i.e., it begins to depend on the level population that varies discretely itself. At the smallest change in the particle size, the collective motion of electrons along the ballistic mode begins. This movement occurs almost without scattering and is characterized by low ohmic losses and high potential, resulting in very high current densities and switching speeds in devices. Electronic energy levels determine the optical and magnetic properties of substance. The changing the particle size makes it possible to control absorption in a wide range of wavelengths. An important aspect of the problem under consideration is also the existence of collective excitations of the states of surface plasmons. All of these effects play an important role in the absorption of electromagnetic energy inside composites.

Stabilization of metal-containing nanoparticles by polymer matrices makes it possible to create composite materials with unique electrical and operational properties [104,120,121,122,123,124,125,126,127,128,129,130]. The development of the science of materials based on metal-containing nanoparticles, including matrix-stabilized polymers, is stimulated by the ever-growing interest in this problem in many areas of chemistry, physics, and materials science. The possibility of combining the properties of a polymer and a metal in one material as well as the regulation of these properties by means of concentration changes has been discussed for a long time. It is known that most metal-containing nanoparticles are thermodynamically unstable. Various polymers can be used to stabilize them, such as polyethylene, polypropylene, polytetrafluoroethylene, and others. These polymers have a relatively high thermal stability, unique rheological properties, and high dielectric strength. They are chemically inert and technologically advanced, which makes it possible to manufacture products of the required shape and size. It is also important that the methods for obtaining these polymers are well-developed.

For the most part, polymeric materials are good dielectrics with stable physical and chemical properties. The chemical stability of polymers allows their use under hard conditions; however, the main application of polymers is as insulating materials. It should be noted that the mechanical properties of polymers can be changed by modifying them with various inorganic fillers. The modification of polymers with specific dielectric properties by carbon nanotubes and metal-containing fillers leads to the creation of composites with increased electrical conductivity compared to the original polymer matrix. The electrical properties of composites depend on the composition, shape, size, and concentration of the filler [120,123].

Various methods are used for the synthesis of metal-polymer composites, such as treatment of polymer films with metal vapors, chemical reactions of metal salts in polymer solutions with subsequent isolation of the corresponding polymer, polymerization of various metal-containing monomer systems, etc. Along with this, the introduction of inorganic fillers into polymers makes it possible to create materials that are characterized by properties of both filler and matrix.

High-pressure polyethylene (HPPE) is most suitable for use as a dielectric matrix for the development of composite materials. The reasons are as follows: the cost of HPPE is not high, the production technology is well-developed, it easy to mix with organic and inorganic fillers, and it belongs to thermoplastic polymers that makes it possible to manufacture products of the required shape and size under mild conditions. All of the above properties of polyethylene have contributed to its intensive use in the electrical industry.

**Figure 2:**
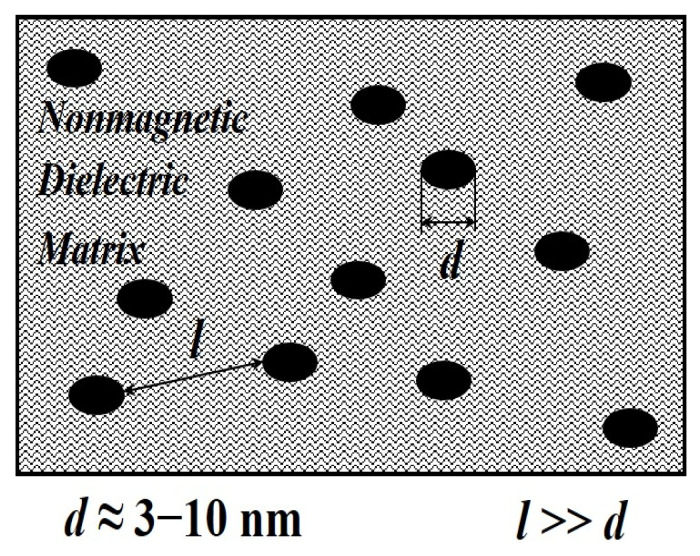
Scheme of nanoparticles stabilization inside a matrix stabilizer.

One of the most promising methods of stabilization is the stabilization of nanoparticles in the solid phase, i.e., the introduction of nanoparticles into matrices of various types. This method is indispensable for obtaining materials whose properties must remain unchanged for a long time. For example, the stabilization of nanosized particles in matrices can find wide application in the production of magnetic materials that can be used as potential devices for recording and storing information or radio-absorbing materials. The general scheme for stabilizing nanoparticles in the volume of stabilizing matrices is shown in Figure 2.

The method of stabilization of nanoparticles with organic polymer matrices was historically the first method for creating nanomaterials [130,131]. Information about the stabilization of metal nanoparticles by various polymer matrices and the approaches used in this case are described in detail in monograph [132]. Note that this method of stabilization is the most developed, and the widespread use of polymeric materials is due to the existence of natural voids in them with sizes necessary for the incorporation and stabilization of nanoparticles of various compositions [133,134].

There are two fundamentally different ways to stabilize nanoparticles using polymer matrices. According to the first method, a polymer suspension or melt is added to the finished dispersion of particles [132]. The second approach is to prepare the dispersion in a polymer medium. The resulting materials differ not only in the most uniform distribution of metal particles in the volume of the polymer but also in the strength of the chemical interaction between the metal-containing nanoparticle and the polymer.

## 4. Electrical and Magnetic Properties of Composite Materials

Of all the variety of physical properties, the most important ones characterizing a substance as a dielectric are electrical properties. These include polarization, electrical conductivity, permittivity, and polarizability of the dielectric. The classification of dielectric substances is based on their most important properties for practice and related functional purposes. Among these substances, there are, for example, piezo- and ferroelectrics, pyroelectrics, electrets, and magneto-dielectrics [135,136].

This section describes the methods used in the study of the electrophysical and magnetic properties of RAM materials and also presents the results of the research:

1. Specific volumetric resistance;
2. Dielectric permittivity in the low-frequency range and in the microwave range;
3. Demagnetization curves by the vibromagnetometer method;
4. Magnetization curves by the Faraday’s method.

### 4.1. Specific Volume Resistance of Composite Materials

#### 4.1.1. Measurement Methodology

**Figure 3:**
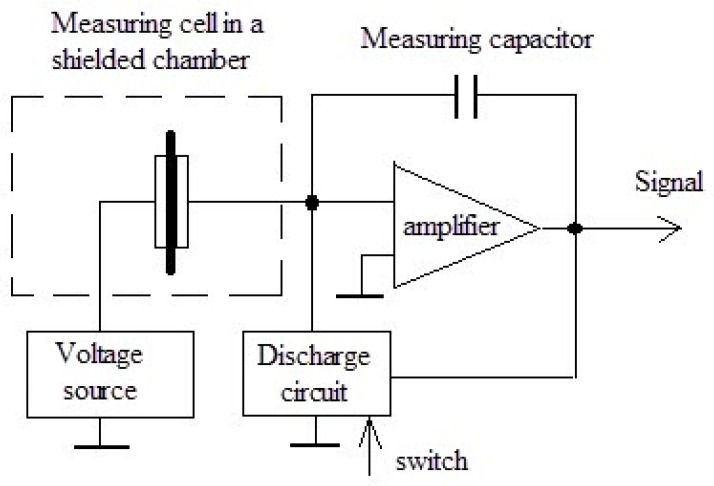
Scheme of a circuit for resistivity measurement. Adapted from [129] with permission.

Measurement of the specific volume resistance *ρ_v_* of synthesized composites was performed by the voltmeter-ammeter method [129,137]. The essence of the method is to measure the currents passing through the sample when a constant electric voltage is applied to it. In this work, the *ρ_v_* measurements of composite materials were carried out according to the two-electrode scheme shown in Figure 3.

The measuring cell is a clamping device with polished brass electrodes with a diameter of 10, 15, 20, and 25 mm and a thickness of 1 mm. The electrodes are isolated from a clamping device by polytetrafluoroethylene insulators. The diameter of the electrode was selected in accordance with the size of the sample in such a way that the possible surface conductivity *ρ_v_* was lower than the measured volume.

The samples under study were disks with a diameter of 20–30 mm and a thickness of 1–3 mm. They had no cracks, dents, chips, burrs, dirt, or scratches visible to the naked eye, and the planes of the samples were parallel. The thickness of the tested samples *L* was determined as the arithmetic mean of measurements at five points evenly spaced over the surface in the intended area of the measuring electrode. The measurements were carried out with a micrometer with an accuracy of 0.01 mm.

The measurement of the resistance *R* of the measuring cell with the sample placed in it was carried out using an electrometric voltmeter, which has a source of measuring voltages in its composition. The cell was placed in a shielded measuring chamber included in the voltmeter kit. The device allowed measuring resistances in the range of 10^3^–10^18^ Ohms at values of measuring voltages 0.1–1000 V.

The measurement process consisted in automatic recording of the readings of the electrometric voltmeter for 20 min with an interval of 1 min. The specific volume resistance of the sample *ρ_v_* (Ohm × m) was calculated by means:

*ρ_v_ = R* × *S/L* (3)

where *R* is the resistance of the sample 20 min after starting measurements (Ohm); *S* is the surface area of the sample covered by the electrode (m^2^); *L* is the thickness of the sample (m).

#### 4.1.2. Experimental Data

**Figure 4:**
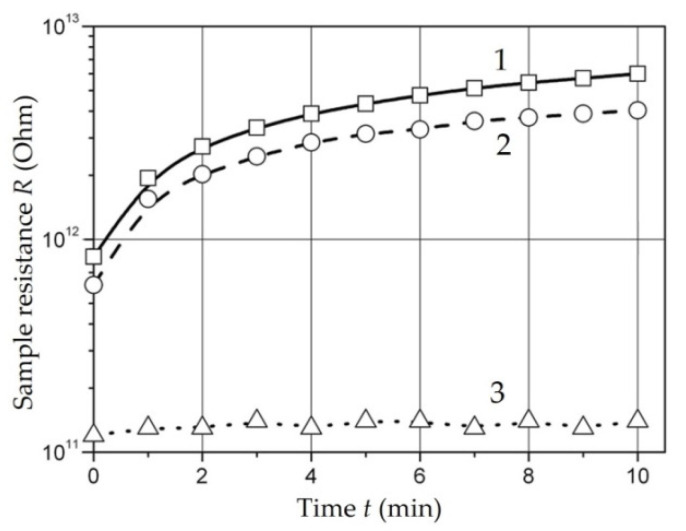
Dependence of the sample *Fe*-03 resistance on time at various operating voltages: 1—10 V; 2—100 V; 3—1000 V. Adapted from [129] with permission.

Figure 4 shows a typical view of the dependence of the resistance on the exposure time under stress for a sample *Fe*-03 with an iron concentration of *C_Fe_* = 20 wt. % synthesized from iron (III) formate [129].

Such dependence of the resistance on the exposure time under voltage is characteristic of polymer dielectrics with ionic electrical conductivity. An increase in the resistance over time can be caused by such reasons as dipole polarization, polarization of the displacement of ions in the sample volume (macro-displacement of ions), and electrical cleaning, which is a decrease in the content of conduction ions in the sample as a result of electrolysis.

*Iron- and Cobalt-Containing Nanocomposites* [129,138,139,140]. The results of measuring the *ρ_V_* of composite materials based on *Fe*- and *Co*-containing nanoparticles are shown in Table 1 [129].

The order of magnitude and the nature of the time dependences of *ρ_V_* allows us to conclude that the electrical conductivity of samples is mainly due to the same mechanisms of ionic and hopping as the electrical conductivity of unfilled polyethylene. The difference between the *ρ_v_* values of unfilled polyethylene and standard values (10^16^–10^18^ Ohm × m) may be due to the increased content of impurities as a result of technological processing.

The resistivity of samples with a filling of 5 and 10 wt. % (samples *Fe*-01 and *Fe*-02) as well as that of unfilled polyethylene weakly depends on the magnitude of the applied field and decreases linearly over the entire range of operating voltages with an increase in the content of nanoparticles.

With an increase in the content of nanoparticles up to 20 wt. % dependence of the specific volume resistance on the applied voltage becomes more pronounced. Thus, in the samples *Fe*-04 and *Co*-01, the specific volume resistance decreases three times and in the sample *Fe*-03 by more than an order of magnitude, with an increase in the applied voltage from 100 to 1000 V.

A decrease in the specific volume resistance of samples when nanoparticles are introduced into the polymer matrix may be the result of a change in the structure of polyethylene towards an increase in amorphousness and porosity, which leads to an increase in molecular mobility and a decrease in the dissociation energy of ions in the polymer. An additional contribution to the conduction current can be made by polarization phenomena caused by the polarizability of nanoparticles: polarization of macro-displacement (within the sample) and micro-displacement (within some areas) as well as slow-forming dipole polarization.

The presence of a tunneling mechanism of electrical conductivity in composites based on metal-containing nanoparticles in a polymer matrix is substantiated [141,142,143]. The presence of this type of electrical conductivity in the material can be determined by the type of current-voltage characteristic (CVC) of the sample. When implementing the tunnel mechanism, the VAC has the form [144]:

*J* = *A* × *V*^2^ × exp(−*B*/*V*) (4)

where *J* is the current density, *V* is the voltage, and *A* and *B* are the constants.

**Figure 5:**
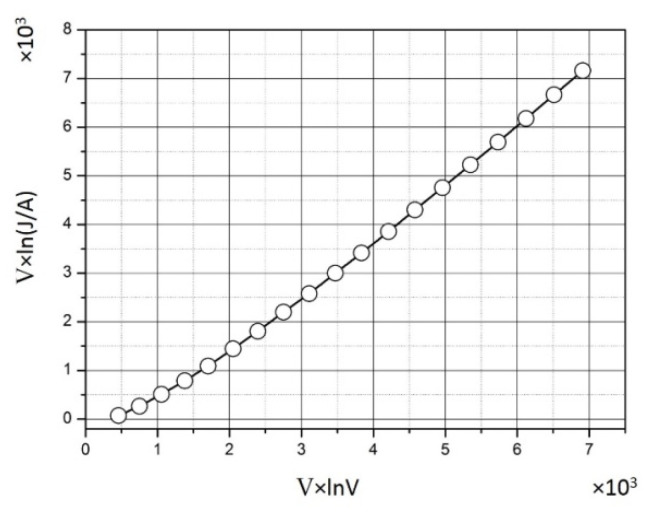
Current-voltage characteristic of a metal polymer based on a polyethylene matrix with iron nanoparticles (about 20 wt. %).

The CVC should have the form of a straight line in Fowler–Nordheim coordinates (*V* × ln(*J/A*); *V* × ln*V*). The sample *Fe*-03 synthesized from *Fe(HCOO)_3_* with *C_Fe_* = 20 mass.% is characterized by the lowest value of *ρ_V_* among the studied samples. For this sample, CVC at a voltage of 100–1000 V was constructed (Figure 5) [129].

According to the type of the obtained CVC, it can be concluded that a tunneling mechanism of electrical conductivity is implemented in the sample *Fe*-03, which contributes to the conduction current.

The electrical conductivity of the filled HPPE is affected in a complex way by both the composition and the average size of the nanoparticles *d*_av_. This is well-illustrated by the lower *ρ_v_* of the sample *Fe*-03 (*d_av_* ≈ 11.5 nm) at a voltage of 1000 V compared with the *ρ_v_* of the sample *Fe*-04 (*d_av_* ≈ 2.4 nm).

The block samples of composites based on iron- and cobalt-containing nanoparticles were obtained by thermal decomposition of formates with *C_Fe_= C_Co_* = 30 wt. % [139]. These samples are characterized by a relatively large particle size (d_cp_ ≈ 8 nm), the presence of a pronounced metallic core, and low values of specific volume resistance (*ρ_v_* ≈ 10^2^ Ohm × m). Such values of electrical conductivity are characteristic of materials having a granular structure with poor contact between granules having their own electronic conductivity, which can also occur in polyethylene containing Fe and Co nanoparticles with a relatively high concentration of metal. The mechanism of electrical conductivity can also be tunnel-like. Such materials, as will be found below, attenuate (shield) the microwave radiation well and, due to the tunneling mechanism of electrical conductivity, can also be used as nonlinear elements of the microwave range [145].

**Figure 6:**
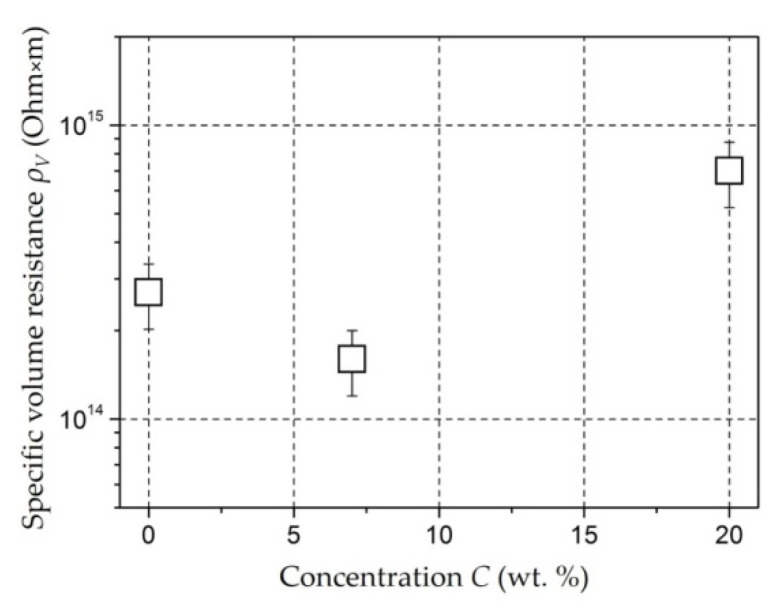
Dependence of resistivity on molybdenum particle concentration *C_Mo_* in a polyethylene matrix.

*Molybdenum-Containing Nanocomposites* [146,147]. The specific volume resistance *ρ_v_* of a composite nanomaterial based on polyethylene containing molybdenum nanoparticles is comparable in order of magnitude (10^14^ Ohm × m) with the value for unfilled polyethylene that has undergone reaction treatment (Figure 6).

This means that no additional electrical conductivity mechanisms arise within the obtained concentrations of the *C_Mo_* filler. Since ionic electrical conductivity is the main mechanism for polyethylene, an increase in the resistivity can be caused by an increase in the dissociation energy of ions under the influence of molybdenum nanoparticles.

*Lead-Containing Nanocomposites* [148,149,150]. Table 2 presents the results of measurements of specific volume resistance *ρ_v_* composite materials based on *Pb*-containing nanoparticles.

The *ρ_v_* values of lead-containing nanocomposites in order of magnitude (10^14^ Ohm × m) coincide with the *ρ_v_* of unfilled HPPE that has undergone reaction treatment. Lead-containing nanocomposites in the order of magnitude (10^14^ Ohm × m) coincide with the *ρ_v_* of unfilled HPPE that has undergone reaction treatment. A slight increase in *ρ_v_* compared to an unfilled matrix may be caused by a decrease in the mobility of charge carriers (molecular ions) when nanoparticles are introduced into the HPPE matrix. In the studied range of filler concentrations, the composite retains the properties of polyethylene as a good dielectric [142].

**Figure 7:**
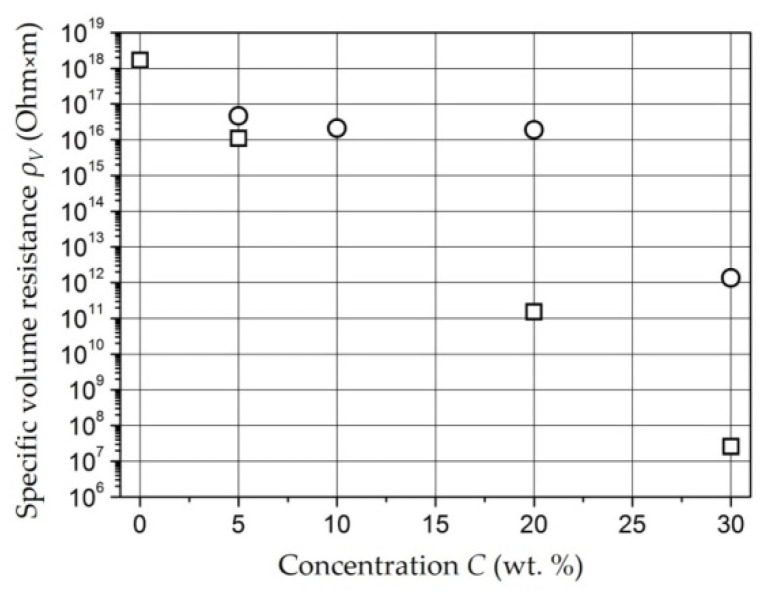
Dependence of resistivity on concentration of *NiFe_2_O_4_* nanoparticles for tablets 0.25 mm thick (squares) and 1.5 mm thick (circles) [151].

*Composites with NiFe_2_O_4_ Nanoparticles* [151,152,153]. With concentration increases, the *ρ_v_* of the samples decrease, but up to a concentration of 20 wt. %, it remains comparable with the *ρ_v_* of unfilled polyethylene (10^14^–10^16^ Ohm × m) (Figure 7) [151]. A noticeable decrease in *ρ_v_* (10^12^ Ohm × m) is observed at concentration of 30 wt. % for a tablet 1.5 mm thick (circles in Figure 7). In a film sample 0.25 mm thick (squares in Figure 7) with concentration of 30 wt. %, an even more noticeable level of conductivity is achieved: 3 × 10^7^ Ohm × m.

The dependence of *ρ_v_* composites based on *Fe*- and *Co*-containing nanoparticles as well as *NiFe_2_O_4_* nanoparticles on the concentration of the metal-containing component is percolation [141,154]. Figure 7 shows that a significant decrease in the resistance of the material occurs at concentrations of 20–30 wt. %.

*Rhenium-Containing Nanocomposites* [124,155,156]. The specific volume resistance of the samples *Re*-02–*Re*-05 in order of magnitude (10^13^ Ohm × m) corresponds to unfilled polyethylene. The exception is the sample *Re*-01, whose specific volume resistance is an order of magnitude lower (10^12^ Ohm × m). This may be a consequence of a higher proportion of components with a low degree of oxidation in rhenium-containing nanoparticles of this sample as well as additional injection of electrons from nanoparticles under the influence of applied voltage (taking into account the fact that the size of *Re*-containing nanoparticles in composites is relatively large at 15 nm) [155].

The studies performed with samples of polymeric composites based on *Bi*, *CeO_2_*, and *CdS* nanoparticles have shown that such composites also retain high values of specific volume resistance for all synthesized concentrations [157,158,159].

### 4.2. Dielectric Permittivity of Composite Materials at Low Frequencies

#### 4.2.1. Measurement Methodology

The determination of the relative permittivity *ε* is based on the dependence of the capacitance of a flat capacitor *C_ε_* on the permittivity of the material filling the space between the electrodes:

*C_ε_* = *ε* × *ε_o_* × *S*/*d* (5)

where *S* is the area of the electrodes of a flat capacitor, *d* is the distance between the electrodes (working gap), *ε* is the relative permittivity of the material filling the working gap of the capacitor, and *ε_o_* is the vacuum electrical constant.

**Figure 8:**
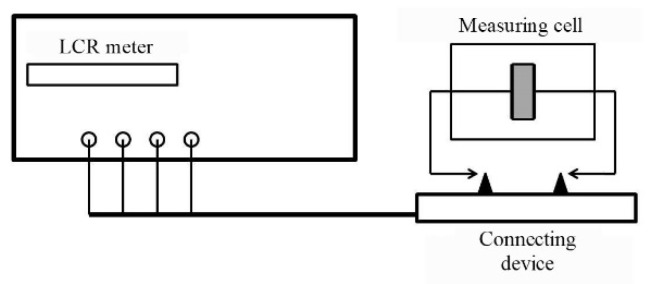
Scheme of the setup for permittivity *ε* measurement.

The measuring cell, a clamping device with polished brass electrodes with a diameter of 10, 15, 20, and 25 mm and a thickness of 1 mm, is a flat capacitor. The measurement of the cell capacity and the tangent of the loss angle (active conductivity) was performed by the bridge method using digital LCR meters E7–8 and E7–12 with operating frequencies *f_op_* = 1 kHz and *f_op_* = 1 MHz, respectively. The measurement error of the capacitance of about 10 pF is 0.3% for both devices. A two-electrode circuit with round electrodes of different diameters was used at the measurements. The block diagram of the measuring unit is shown in Figure 8.

In according with the measured capacity of the sample cell:

*C_x_* = *C_ε_* + *C_p_*, (6)

where *C_x_* is measured capacitance, *C_p_* is correction consisting of the sum of the parasitic capacitance and the lateral capacitance of the measuring capacitor, and the geometric capacitance of a flat capacitor *C_ε_* filled with the material under study was calculated.

The correction *C_p_* was preliminarily calculated from the measured values of the capacitance of the measuring capacitor with air filling or by formulas that take into account the geometry of the measuring capacitor [160].

The value of the dielectric constant *ε* is calculated by the formula:

*ε* = *C_ε_* × *d*/(*ε_0_* × *S*) = (*C_x_*− *C_p_*) × *d*/(*ε_0_* × *S*), (7)

where *d*, *C_p_*, *C_x_*, and *S*= *πd*^2^/4 are the measured values.

The tangent of the loss angle of the material under study is calculated by the formula:

(8) $$\tan h(\delta_{\varepsilon })=\tan h(\delta_{x})\times \frac{C_{x}}{\left(C_{x}-C_{p}\right)},$$

where tanh(*δ_x_*) = *G_x_*/(*ω* × *C_x_*) is the measured value of the tangent of the loss angle of the measuring capacitor.

The samples under study are similar to those described in Section 4.1 of this work.

The dielectric permittivity of various samples of composite materials based on metal-containing nanoparticles stabilized in a HPPE matrix was measured using the above method. The results of these measurements and their interpretation are presented below.

#### 4.2.2. Experimental Data

*Iron- and Cobalt-Containing Nanocomposites* [129,138,139,140]. The results of the dielectric permittivity measurement are presented in Table 3 [129]. The samples based on cobalt-containing particles with *C_Co_* = 30 wt. % were synthesized from cobalt formate, and the samples were pressed at different temperatures: *Co*-02 at 230 °C and *Co*-03 at 280 °C.

The monotonic increase in ε with increasing *C_Fe_* is due to the contribution to the total polarization of the polarization of *Fe*- and *Co*-containing nanoparticles, whose polarizability is higher than the polarizability of the matrix due to the higher mobility of the electron shells. The difference in the values of the dielectric permittivity of the *Fe*-03 and *Fe*-04 samples with the same concentration of iron-containing nanoparticles is associated with a higher susceptibility of large nanoparticles. The high dielectric permittivity of the *Co*-02 and *Co*-03 samples may be associated with a percolation transition in a granular nanocomposite [142].

**Figure 9:**
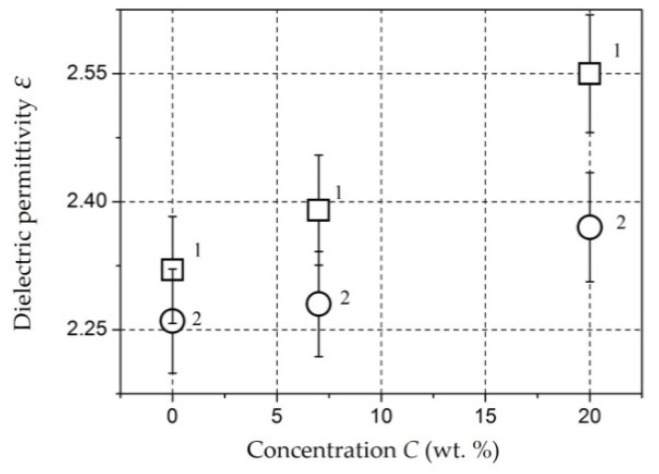
The dependence of the permittivity ε of composite nanomaterial on the mass concentration of Mo in HPPE: 1, at a frequency of 1 kHz; 2, at a frequency of 1 MHz. Adapted from [146] with permission.

*Molybdenum-Containing Nanocomposites* [146,147]. The results of measuring the permittivity of composite materials based on Mo-containing nanoparticles are shown in Figure 9 [146]. The monotonous increase in the permittivity ε is caused by an increase in the contribution of the polarization of nanoparticles to the total polarization of the composite with an increase in the mass fraction of the filler [146]. However, for molybdenum-containing composite materials, an increase in the dielectric constant relative to unfilled polyethylene at *C* up to 20 wt. % is insignificant.

*Lead-Containing Nanocomposites* [148,149,150]. The results of the measurements of *ε* of Pb-containing composites at frequencies of 1 kHz and 1 MHz are presented in Table 4 [150].

The nonmonotonic behavior of the dielectric permittivity with an increase in the mass content of nanoparticles looks unexpected. The contribution from the polarization of nanoparticles usually leads to an increase in *ε* [129]. The weak polarizability of lead-containing nanoparticles may be related to the peculiarities of their phase state (liquid–solid) and requires additional study [150].

**Figure 10:**
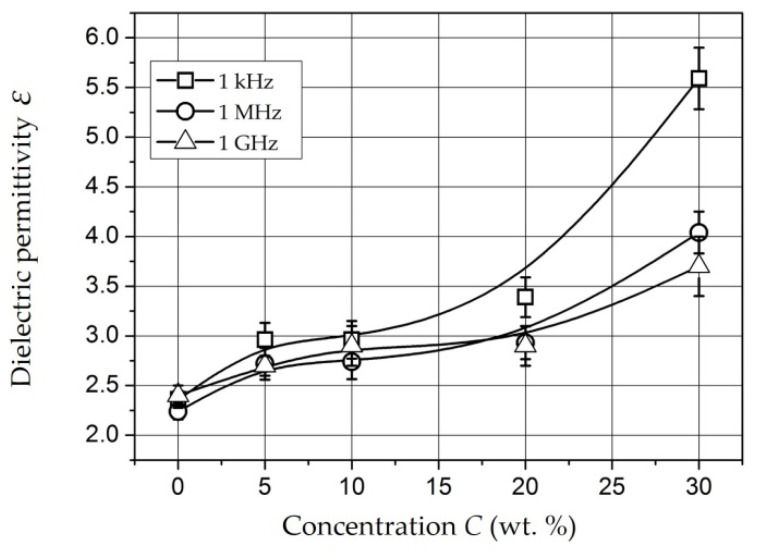
Dependence of dielectric permittivity ε on concentration of NiFe_2_O_4_ nanoparticlesat a frequency of 1 kHz (squares), 1 MHz (circles), and 1 GHz (triangles) [151].

*Composites with NiFe_2_O_4_ nanoparticles* [151,152,153]. The dependencies of dielectric permittivity of samples on *NiFe_2_O_4_* nanoparticlesare presented in Figure 10.

The samples under study were tablets of 1.5 mm thick. The dependencies were measured at a frequency of 1 kHz (squares), 1 MHz (circles), and 1 GHz (triangles). It can be seen that permittivity increases with increasing concentration and, changes relatively weakly at *C* = 5–20 wt. % (*ε* = 2.7–3.4). At *C* = 30 wt. %, it reaches values of 5.6 (for 1 kHz) and 4 (for 1 MHz). This dependence of *ε*(*C*) may also indicate the presence of a percolation transition in composites based on *NiFe_2_O_4_* nanoparticles in the concentration range of 20–30 wt. % [151].

*Rhenium-Containing Nanocomposites* [124,155,156]. The results of measuring the dielectric permittivity of *Re*-containing composites are presented in Table 5 [124].

The difference between the *ε* of composites with Re-containing nanoparticles from the corresponding values for unfilled polyethylene that has undergone reaction treatment may be caused by differences in the component composition of nanoparticles. A higher content of rhenium compounds with a low degree of oxidation (*Re*, *ReO_2_*, *ReO_3_*) leads to higher mobility of electron shells and polarizability of nanoparticles. The consequence of this is higher values of *ε* for samples *Re*-01 and *Re*-04. Conversely, the predominance of compounds with a high degree of oxidation (*Re_2_O_7_*) does not give a noticeable additional contribution to the polarization of the composite and dielectric losses. Therefore, the values of *ε* for the *Re*-02 sample differ slightly from the *ε* of unfilled polyethylene [155,156].

### 4.3. Investigation of the Properties of Composite Materials Using a Measuring Line

**Figure 11:**
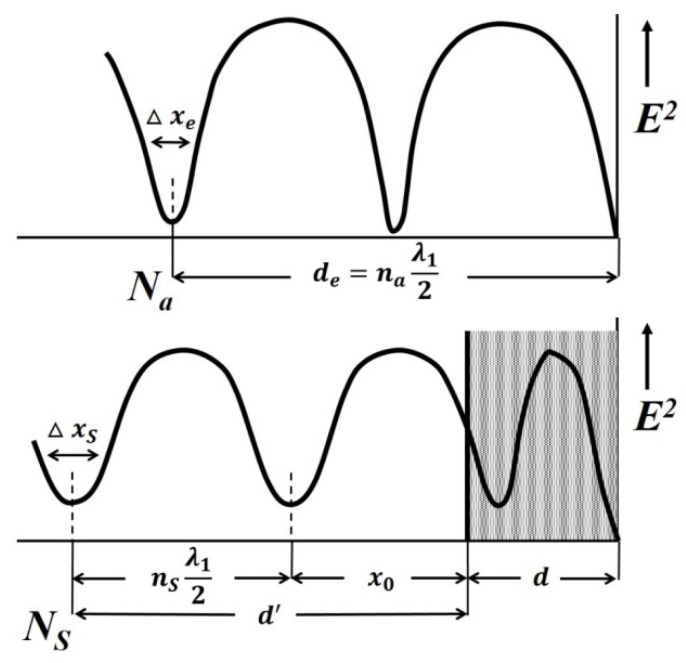
Standing waves in a short-circuited waveguide (upper) and in a short-circuited waveguide with a sample located in it (lower). Adapted from [129] with permission.

The dielectric permittivity measurement by means of the closed-loop line method is based on observing the pattern of standing waves in the waveguide path containing the sample under study (Figure 11).

This method is most common when measuring the dielectric properties of materials since, in comparison with other methods in many practical cases it turns out to be relatively simpler and more universal in terms of the technique of preparing and conducting the experiment. Its disadvantage should include more cumbersome calculations to obtain the final results. The test sample of the material is placed in a segment of a rectangular waveguide attached to the measuring line. The sample must completely fill the section of the waveguide and fit snugly to the short-circuiting flange. In principle, the length of the sample can be arbitrary [160].

The measurements were carried out in the frequency band 20–53 GHz. This range was overlapped by three bands. For each band, special standard measuring equipment is used (Table 6) [129].

**Figure 12:**
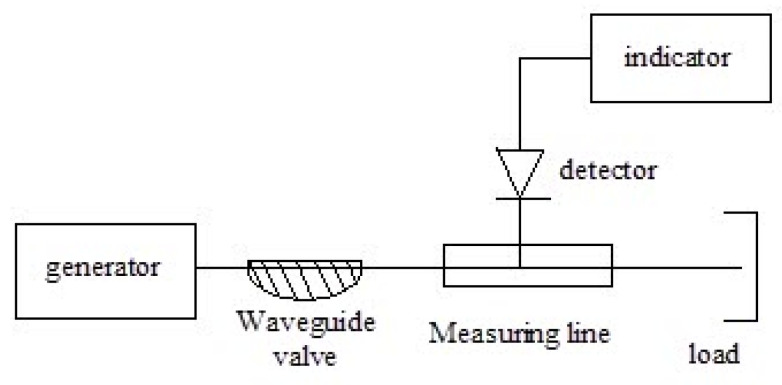
Scheme of the setup for the study of dielectrics in the microwave range using a measuring line. Adapted from [129] with permission.

The scheme of the setup used is shown in Figure 12. The generator signal is modulated by a 1 kHz meander and, after quadratic detection enters the input of a selective microvoltmeter B6–9. The output of it is connected with a digital voltmeter, B7–38. The measuring line has a precise movement mechanism that allows one to determine the position of the probe with an error of 0.01 mm.

The measurement principle is based on determining the reflection coefficient (or impedance) at the input of a short-circuited line segment filled with the sample under study.

**Figure 13:**
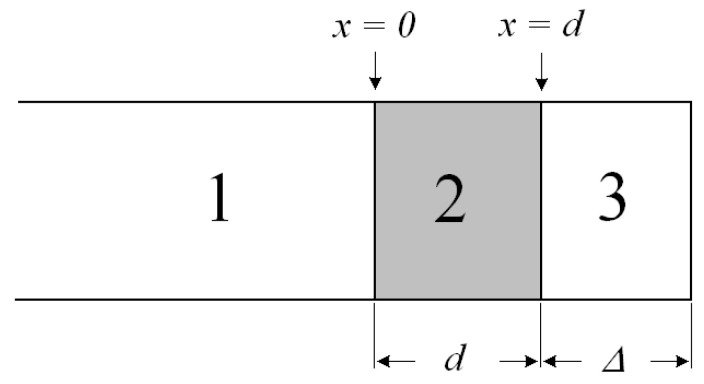
The scheme of inclusion of the sample.

The scheme of inclusion of a sample with thickness *d* in the waveguide path and the picture of standing waves are shown in Figure 13.

An electromagnetic wave falls on the interface with the sample 2 from medium 1 (empty waveguide). The standing wave in medium 1 is the result of the interference of the incident wave and the waves obtained as a result of reflection from the interface (*x* = 0) and the short-circuit wall. The short-circuit wall can be in two positions: *x* = *d* (case of short circuit) or *x* = *d* + *Δ* (case of idling).

The use of a measuring line allows measurements to be made in two positions: short circuit and idle (open line). The equivalent of an open line is a quarter-wave section formed by a movable short-circuited load located behind the sample.

The total resistance in the cross section *x* = 0 from the medium 1 can be defined for each position aforementioned by measuring the wavelength in an unfilled waveguide *λ_1_*, the voltage standing wave coefficient (VSWC) *K_swU_* = *E_max_*/*E_min_*, and the distance from the boundary of the dielectric to the first node of the standing wave in the direction of the generator *x*_0_:

(9) $$Z\left(0\right)=Z_{1}\times \frac{1-i\times \frac{E_{max}}{E_{min}}\times \tan h\frac{2\pi x_{0}}{\lambda_{1}}}{\frac{E_{max}}{E_{min}}-i\times \tan h\frac{2\pi x_{0}}{\lambda_{1}}}$$

where $Z_{1}=\frac{2b}{a}\times 120\pi \frac{\lambda_{1}}{\lambda_{0}}$ is the wave resistance of an unfilled waveguide, *a* and *b* are the dimensions of the wide and narrow walls of the waveguide, respectively, *i* is imaginary unit.

The total resistance in cross-section *x* = 0 from the medium 2 are equal to:

- *Z*(0) = *Z_2_* × tanh(*γ_2_d*), where *Z_2_* and *γ_2_* are the wave resistance and propagation constant of the waveguide filled with the material under study, respectively, in the case of a short circuit (*Δ* = 0);
- *Z*(0) = *Z_2_* × cth(*γ*_2_*d*) in the case of idling (*Δ* = *λ_1_*/4).

As a result, complex equations are obtained for determination of the parameters of the waveguide filled with the material under study, *Z_2_* and *γ_2_*:

$$\frac{Z_{2}}{Z_{1}}\times \tan h\left(\gamma_{2}d\right)=\frac{1-i\times \frac{E_{max}}{E_{min}}\times \;\tan h\frac{2\pi x_{0}}{\lambda_{1}}}{\frac{E_{max}}{E_{min}}-i\times \;\tan h\frac{2\pi x_{0}}{\lambda_{1}}}\;—\mathrm{in}\;\mathrm{case}\;\mathrm{of}\;a\;\mathrm{short}\;\mathrm{circuit};\;\frac{Z_{2}}{Z_{1}}\times coth\left(\gamma_{2}d\right)=\frac{1-i\times \frac{E_{max}}{E_{min}}\times \;\tan h\frac{2\pi x_{0}}{\lambda_{1}}}{\frac{E_{max}}{E_{min}}-i\times \;\tan h\frac{2\pi x_{0}}{\lambda_{1}}}\;—\mathrm{in}\;\mathrm{case}\;\mathrm{of}\;\mathrm{idling}.$$

Hence, the measured values of the VSWC and *x_0_* for cases of short circuit and idling will be different.

The samples *Fe*-01 with 5 wt. % of *Fe(CO)_5_*, *Fe*-02 with 10 wt. % of *Fe(CO)_5_*, and *Fe*-03 with 20 wt. % of *Fe(HCOO)_3_* were examined [129].

**Figure 14:**
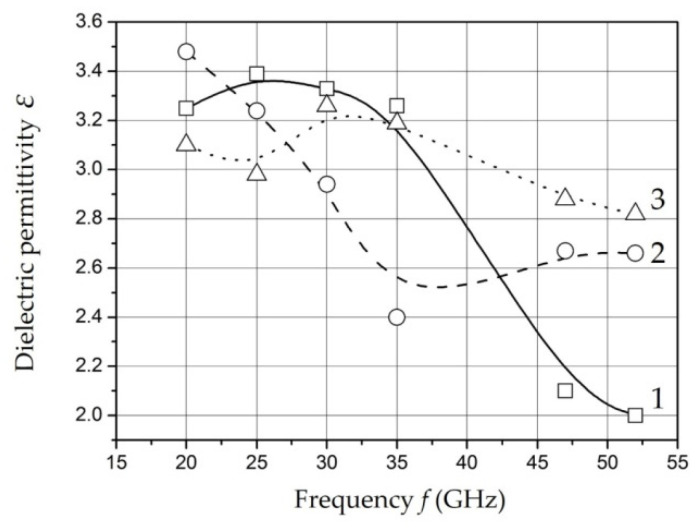
The dependence of the dielectric permittivity of the samples *Fe*-01 (1), *Fe*-02 (2), and *Fe*-03 (3) on the operating frequency. Adapted from [129] with permission.

**Figure 15:**
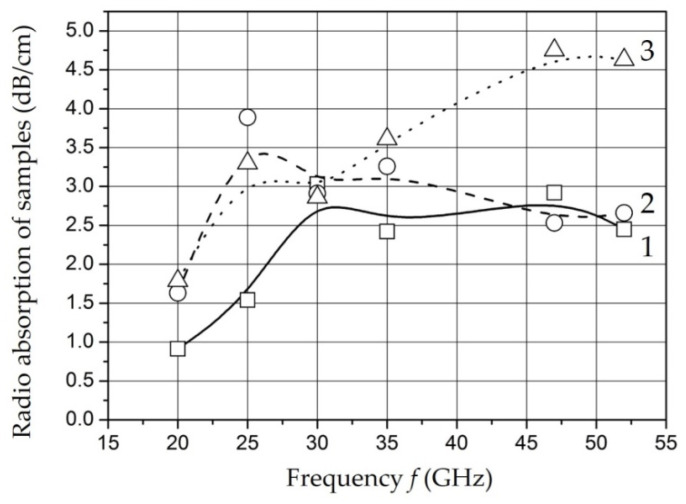
The dependence of the radio absorption of the samples *Fe*-01 (1), *Fe*-02 (2), and *Fe*-03 (3) on the operating frequency. Adapted from [129] with permission.

The results of measurements of the dielectric permittivity and water absorption of the samples with different mass filling in the microwave range are shown in Figure 14 and Figure 15 [129].

The ratio of amplitude and time characteristics of relaxation processes can be estimated in according with measurements of the dielectric permittivity and radio absorption of samples in the microwave range. The decrease in dielectric permittivity with a relatively constant level of losses in the operating frequency range for the samples *Fe*-01 and *Fe*-02 can be explained by a decrease in the intensity of relaxation processes while maintaining relaxation time. An increase in the content of nanoparticles to 20 wt. % (sample *Fe*-03) is accompanied by a decrease in relaxation time at a relatively constant intensity.

It should be noted that there is a tendency to decrease the time and increase the intensity of the relaxation process of dipoles with an increase in the content of nanoparticles in the polymer. The obtained dependencies can be associated with the polarization of metal-containing nanoparticles as well as for low frequencies. It is known that its polarizability increases with size of nanoparticles increase [161]. The results of microwave measurements are characterized in the studied samples as materials having uniform absorption and dielectric permittivity parameters in a wide band of the studied frequency range.

### 4.4. Investigation of the Properties of Composite Materials by the Resonance Method

The study of the properties of materials using coaxial resonators with an end gap is based on determination of the shift of the resonant frequency and the change in the quality factor of the resonator in the presence of a sample under study [162,163]. The method of placing the sample in the resonator depends on what characteristics of the material (permittivity, tangent of the dielectric loss angle, surface impedance, etc.) are going to be measured. The dielectric constant ε and the tangent of the dielectric loss angle tanδ of polymeric composite materials were studied [104,146,151,164,165,166,167,168].

**Figure 16:**
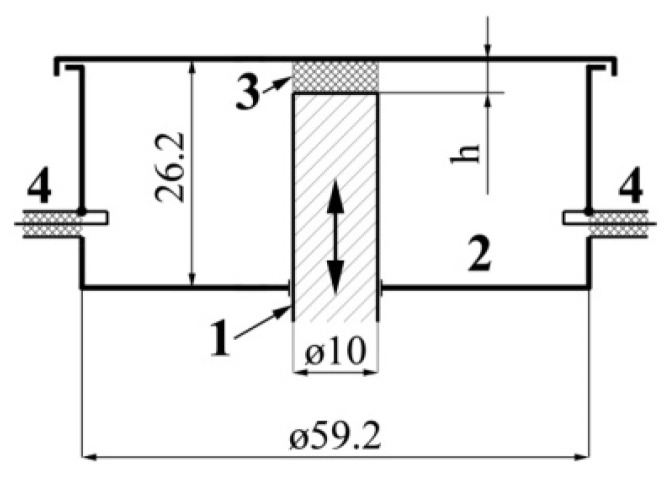
The scheme of a tunable coaxial resonator with an end capacitive gap.

The scheme of an experimental setup based on a measuring coaxial resonator with an end gap is shown in Figure 16.

**Figure 17:**
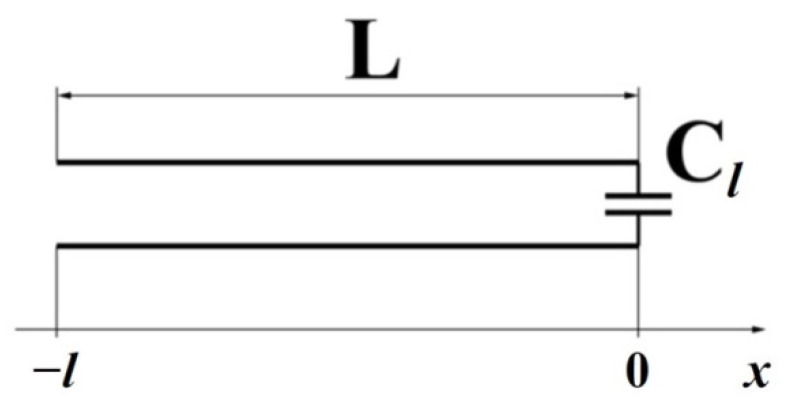
An equivalent scheme of a resonator used.

The brass resonator is consisted of a movable central rod 1 with a closed cylindrical working volume 2 placed on the axis of 1. The rod is moved by a backlash-free micrometric mechanism equipped with a vernier position indicator. All surfaces inside the working volume are polished and have a silver coating. The capacitive gap 3 is designed for tuning into resonance and for placing samples under study inside it. Its height h can be changed by moving the movable central conductor. The resonator is connected to the signal generator and the spectrum analyzer by means of inductive communication loops 4. The dimensions of the resonator parts used in calculations are presented in Figure 16. An equivalent circuit of such a resonator is a long line with a capacitive load (Figure 17).

The resonant frequency of such a circuit can be defined by means the total complex resistance of the long line at its input (in section with the coordinate *x* = −*l*:

(10) $$Z_{in}\left(-l\right)=Z_{w}\times \frac{Z_{l}+Z_{w}\times \tan h(\gamma l)}{Z_{w}+Z_{l}\times \tan h(\gamma l)}$$

where *Z_w_* and *Z_l_* are wave resistance of the line and resistance of the load, respectively, *γ = α + iβ* and is *l = L* are the electromagnetic wave propagation constant in the line and length of the line, respectively.

For a line with negligible attenuation (*α* = 0), the relation (10) has the form:

(11) $$Z_{in}\left(-l\right)=Z_{w}\times \frac{Z_{l}+i\times Z_{w}\times \tan h(\beta l)}{Z_{w}+i\times Z_{l}\times \tan h(\beta l)}$$

The zero input impedance of the line *Z_in_*(–*l*) = 0 is the resonance condition. Thus,

*Z*_l_ + *i* × *Z_b_* × tanh(*βl*) = 0. (12)

Taking into account that, for a capacitive load *Z_l_* = 1/(*i* × *ω_r_* × *C_l_*), and also *β* = *ω_r_*/*c* for an air-filled coaxial line, a nonlinear equation for finding the resonant frequency of a coaxial resonator with an end gap can be obtained:

1/(*ω_r_* × *C_l_*) = *Z_w_* × tanh(*ω_r_* × *C_l_*/*c*) (13)

where *c* is the velocity of light in vacuum.

The measurement method has the next algorithm. First, by varying the frequency of the generator, the system is tuned into resonance, and the resonant frequency *f_r_* and quality factor *Q_0_* of the resonator are measured without a sample. Then, a sample is placed in the resonator, and the same measurements are carried out again. The resonance frequency *f_r_’* and the quality factor *Q’* of the resonator with the sample are obtained.

**Figure 18:**
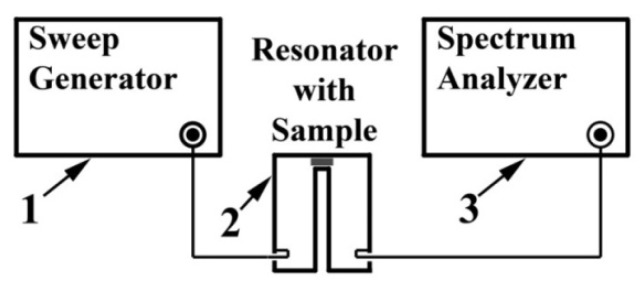
The scheme of an experimental setup for measuring *ε* and tanh(*δ*) in a coaxial resonator with an end gap.

The scheme of the measuring unit is shown in Figure 18. A signal generator (SME06, Rohde&Schwartz) with operating frequency range of 10 kHz–6 GHz was used as a signal source 1. A spectrum analyzer (FSP7, Rohde&Schwartz) 3, with an upper limit frequency of 7 GHz together with generator 1, makes it possible to observe the resonant curve of the measuring resonator 2 on the screen and automatically perform frequency and amplitude measurements necessary to determine the resonant frequency and quality factor.

**Figure 19:**
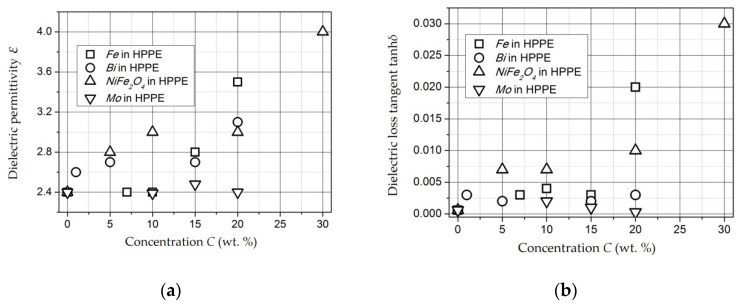
Dependence of dielectric permittivity (**a**) and dielectric losses (**b**) of nanocomposites on concentration of the nanoparticles *Fe*, *Bi*, *NiFe_2_O_4_*, and *Mo*.

This method was used to study the concentration dependences of composite materials based on *Fe*-, *Bi*-, and *Mo*-containing nanoparticles as well as *NiFe_2_O_4_* nanoparticles in a HPPE matrix [165]. The results of measurements of *ε* and tanh(*δ*) for samples with *Fe*, *Bi*, *Mo*, and *NiFe_2_O_4_* nanoparticles in a HPPE matrix are presented in Figure 19. The analysis of concentration dependences allows us to conclude that there are percolation transitions in the concentration range of 15–20 wt. % for samples with *Fe*-containing nanoparticles and in the range of 20–30 wt. % for samples with *NiFe_2_O_4_* nanoparticles. In the case of *Fe*-containing nanoparticles in the ultra-high-frequency range, a shift of the percolation transition to the region of lower concentrations is observed.

The dielectric permittivity of Bi-containing composites also monotonically increases with increasing concentration of the nanoparticles, while tan*δ* is slightly higher than that of unfilled polyethylene and remains constant up to *C_Bi_* = 20 wt. %. *ε* and tanh(*δ*) of *Mo*-containing composites in the studied concentration range differ slightly from the values of *ε* and tanh(*δ*) of the unfilled matrix.

It should also be noted that the increased values of dielectric permittivity relative to unfilled polyethylene remain in the microwave range. This means that the value of *ε* of the composite materials based on a nonpolar polyethylene matrix is determined by electronic polarization.

### 4.5. Magnetic Properties of Composite Materials with Ferromagnetic Nanoparticles

Synthesis and research of nanoparticles with high values of coercive force is an important task on the way to creating new magnetic materials. However, the manufacture of materials from nanoparticles leads to their agglomeration and loss of properties characteristic of individual nanoparticles. Thus, there is a need to create composite materials based on polymers with metal-containing nanoparticles localized into the volume of the matrix. The resulting composite materials are possessed most of the properties inherent in polymers, while the magnetic properties of nanoparticles are preserved [169,170,171]. Polymer composites with magnetic nanoparticles are of particular interest for the development of radio-absorbing materials and coatings. It could be possible since on their basis, it is possible to implement bicomplex electrodynamic media with the complex permittivity (*ε*) and permeability (*µ*) differing from the unit. Combining ferromagnetic inclusions with inclusions of other types, it is possible to create the desired set of effective broadband radio-absorbing coatings [172,173,174,175,176,177,178,179,180,181,182].

**Figure 20:**
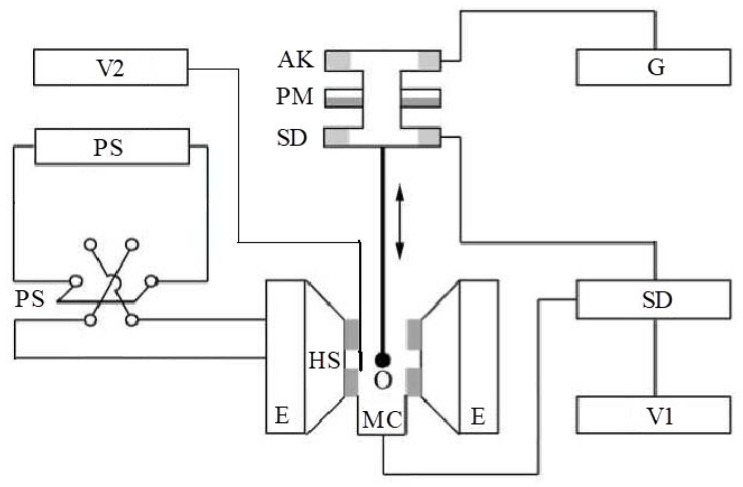
Electrical scheme of a vibrating magnetometer: PS is a electromagnet power supply with a polarity switch; HS is a Hall sensor; E is an electromagnet; MC are the measuring coils; AK is a coil for measuring amplitude; PM is a permanent magnet; G is a generator; SD is a synchro detector; V1 and V2 are the voltmeters; O is a sample.

Let us consider the magnetic characteristics of the obtained composite materials on the example of *NiFe_2_O_4_* and *Fe*- and *Co*-containing nanoparticles stabilized in the volume of a high-pressure polyethylene matrix [151,173,181,182]. Studies of composite materials containing these ferromagnetic nanoparticles were carried out by the vibration magnetometer method. This technique is based on an induction method for measuring magnetic properties. A scheme of a vibration magnetometer is presented in Figure 20. A sample O mounted on a rod is set in oscillatory motion in a system of four measuring coils. In this case, the oscillation axis is parallel to the plane of the coils, and the magnetic moment of the sample, induced by an external magnetic field, is oriented perpendicular to the plane of the coils. The coils are located at the poles of the electromagnet (E) sourcing magnetic field. The electromagnetic field (EMF) in the coils is determined by the flux coupling with the sample and depends not only on the magnetic moment of the sample but also on the geometry of the coils and the size and shape of the sample. Therefore, direct measurement of the absolute values of the magnetic moment (magnetization) is difficult. As a rule, a comparison method is used with a reference sample, which has a size and shape close to the sample under study and known magnetic characteristics.

By means of the induction measurement method, only the magnetic moments of the samples can be directly compared. In order to move on to specific characteristics (magnetization *M* or specific magnetization *σ*), it is necessary to know the volumes or masses of the samples. Magnetization is the magnetic moment of a unit of volume. It has the dimension (G) or (A/m). Specific magnetization is the magnetic moment of a unit of mass. It has the dimension (G × cm^3^/g) or (emu/g) or (A × m^2^/kg). These characteristics are interconnected through the material density *ρ*:

*M* = *ρ* × *σ* (14)

It is easier to determine the mass of samples from experiment. Therefore, in comparative measurements, specific magnetization is more often used.

In works [151,173,181], the studies were carried out by means of a vibrating magnetometer (PAR-155, EG&G) with an absolute sensitivity of 10^−5^ emu. The noise level at the integration time of 1 s and 3 s were 5 × 10^−5^ emu and 2 × 10^−5^ emu, respectively. The diameter of the electromagnet poles and the distance between poles were 60 mm and 50 mm, respectively. The value of the field in the gap can be 1.2 T at a current of 22 A. As part of the setup, a power supply unit was used to provide the maximum current values at the level of 8 A; therefore, the maximum field values were limited to 0.5 T. It was usually sufficient for the studied compositions. Magnetic field was measured by means of a Hall sensor. Power to a Hall sensor was supplied from a precision 100 mA current source. The potential was measured by a voltmeter having a sensitivity of 0.1 µV. The final field resolution was 0.01 G. The relative error did not exceed 0.5%. A helium cryostat with the temperature range 4.2–300 K was used for measurements at low temperatures. The temperature was measured and stabilized using a temperature controller. The accuracy of temperature stabilization was 0.1 K at temperatures below 100 K and not worse than 0.2 K for temperatures of 100–300 K.

The sample size could not exceed 3 mm due to cryostat restrictions. The measuring error in the magnetic moment associated with the size and shape of the sample did not exceed 1% for such sample sizes. The magnetometer was calibrated according to a nickel sample at room temperature.

**Figure 21:**
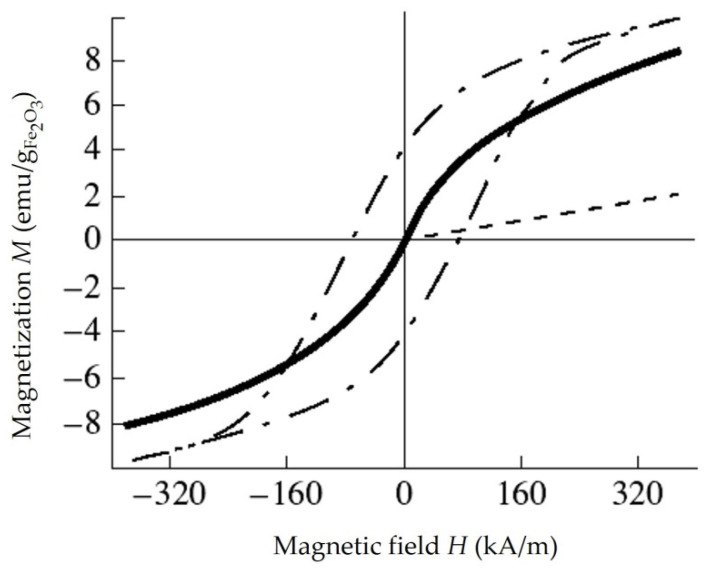
Experimental dependencies of magnetization *M* on magnetic field *H* for a sample containing 5 mass. % *γ-Fe_2_O_3_* in a polyethylene matrix at a temperature of 293 K. The dotted line and dotted line with dots correspond to paramagnetism and ferromagnetism, respectively. Adapted from [181] with permission.

*Iron-Containing Composites* [173,181]. The experimental dependence of the magnetization *M* on the magnetic field *H* for a sample containing 5 mass. % *γ-Fe_2_O_3_* in a polyethylene matrix at room temperature (293 K) is presented in Figure 21 [181].

It can be seen that the experimental dependence of magnetization *M*(*H*) is nonlinear and does not detect saturation in the 4.7 kOe field, nor does any hysteresis occur on it. The shape of the curve *M*(*H*) indicates that the system of magnetic particles *γ-Fe_2_O_3_* in the sample at room temperature is in a superparamagnetic state. Similar results were obtained at other concentrations of *γ-Fe_2_O_3_* in the HPPE matrix.

In addition to the above measurements, the effect of temperature on the magnetic properties of the obtained composite materials consisting of a matrix of high-pressure polyethylene and iron-containing nanoparticles synthesized by decomposition of iron pentacarbonyl was studied. The studied samples were heated for 10 min in air at temperatures of 195 °C, 215 °C, 240 °C, 260 °C, and 290 °C. At each temperature, six samples obtained by the same method were heated, and all of them were examined in order to exclude experimental error. After warming up, three samples from each series were magnetized and cooled in a magnetic field with the strength of 7 kOe, which we will call the “texturing” field. The remaining three samples were examined without processing by a “texturing” field.

For samples cooled in a magnetic field, magnetization loops were measured both along and across the direction of the “texturing” magnetic field. As a result of the studies, no difference was found in the magnetization curves of “textured” and non-”textured” samples in the magnetic field. It was typical for all series of samples subjected to temperature treatment.

Such behavior of samples containing a magnetic component in relation to the directed magnetic field applied to them can be explained by two reasons. The first is the formation of strong chemical bonds between the matrix and metal nanoparticles localized in it, and the second is the small contribution of the magnetic anisotropy of the shape to the overall magnetic anisotropy of the particles.

**Figure 22:**
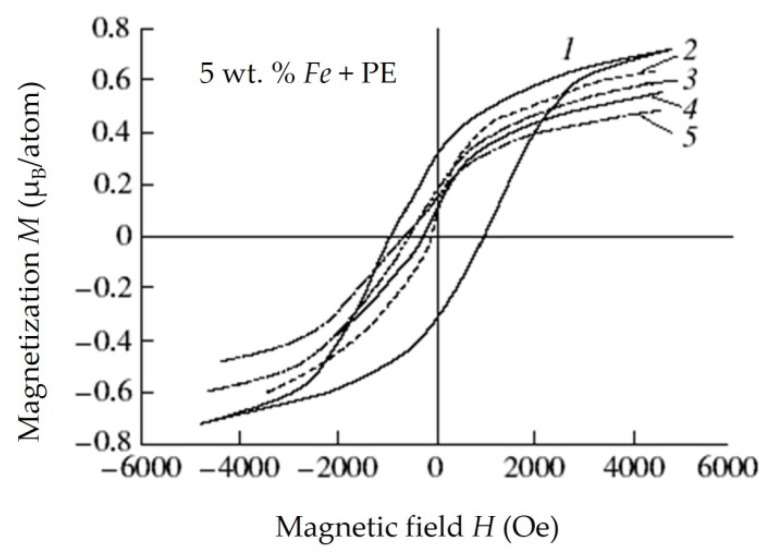
Hysteresis loop of a sample containing 5 mass. % of iron-containing nanoparticles in the volume of the polyethylene matrix: the initial sample (1) and demagnetization loops for air-heated samples at (2) 29 °C, (3) 240 °C, (4) 260 °C, and (5) 195 °C.

The hysteresis loop of the initial sample and the demagnetization curves of the entire series of heat-treated samples are shown in Figure 22 [173]. The corresponding values of the residual magnetization of samples and samples placed in a magnetic field with the strength of 4.5 kOe as well as their coercive force at room temperature and at temperature of 100 °C are presented in Table 7.

During the research, it was noted that even at a temperature of 240–260 °C, when the polyethylene melts, a coercive force is observed in the sample. However, compared to the initial sample, it is much smaller. This can be explained by the formation of *γ-Fe_2_O_3_* due to the oxidation of the metal phase in nanoparticles. Iron (III) oxide has a lower magnetocrystalline anisotropy compared to metallic iron, which is the base component in the initial composite material [181].

A specific behavior of residual magnetization was observed for all the studied samples that underwent heat treatment. However, one trend can be noted that, with an increase in the calcination temperature of the samples, there was a decrease in the magnitude of their residual magnetization, and a decrease in the magnitude of the coercive force of the materials studied was also noted. However, when the samples were magnetized in a magnetic field with strength of 4.5 kOe, a decrease in the magnitude of magnetization was observed at first and then its increase. This feature is most likely related to the redox processes that occurs when the composite material is heated and the partial destruction of the matrix material on the basis of which the composite is obtained.

According to the data of Mossbauer spectroscopy, the following transformations occur with nanoparticles in synthesized materials. Initially, the oxidation of nanoparticles to iron (III) oxide occurs, a further increase in temperature leads to the polymer matrix starting to collapse, resulting in the reduction of iron (III) oxide to *Fe_3_O_4_*. Such a heat treatment process at temperatures close to the destruction temperature of the matrix can be successfully used to increase the residual magnetization in samples [181].

The study of the temperature behavior of magnetization was carried out for a sample that was first calcined at a temperature of 210 °C for 10 min and then underwent additionally 60 min calcination. According to the data obtained, it was noted that additional calcination leads to a decrease in the coercive force by about 5%. It may be associated with the oxidation of nanoparticles.

The effect of low temperatures on the magnetic properties of synthesized iron-containing composites was also investigated. The nanomaterials containing 5 wt. % of iron-containing nanoparticles in the volume of the polyethylene matrix (having a coercive force of 950 Oe) were investigated. The effect was carried out in a cyclic mode. Initially, the sample was cooled to −70 °C, kept at this temperature for 2 h, and then quickly heated to a temperature of +60 °C for 2 min and kept at the same temperature for 30 min and then quickly frozen again. Five such cycles with each sample were carried out. Along with this, a series of samples were kept only at −70 °C for 9 h, and three samples were kept for 21 h. Such temperature effects did not have a significant effect on the coercive force of the studied nanomaterials, and its changes amounted to 30–50 Oe.

*Cobalt-Containing Nanocomposites.* Among the works devoted to materials with nanoparticles of magnetic metals, after traditional iron, cobalt occupies the second place [169,179,182]. Cobalt in the form of samples of ordinary (macro) sizes is a ferromagnetic metal with a Curie temperature of *T_C,b_* = 1390 K. This is the highest value for existing magnetic materials [183]. It characterized by a saturation magnetization of *M*_S,b_ = 1.79 T at room temperature and coercive force *H_c,b_* = 10 Oe [184,185]. The hexagonal *α*-phase of cobalt is stable at temperatures below 420 °C. The cubic *β*-phase of cobalt is stable above 420 °C. The crystal structures of α- and β-phases are close, and their saturation magnetizations also almost coincide. The magnetic anisotropy for the cubic modification is less than for the hexagonal one. It is of *K_v_* = 2.5 × 10^5^ J/m^3^, 7.5 × 10^4^ J/m^3^, or 2.5 × 10^4^ J/m^3^ according to data from [186,187,188], [189], or [190,191], respectively. For the hexagonal phase, *K_v_* = 4.5 × 10^5^ J/m^3^ [192]. In most of the synthesis methods used today, cobalt nanoparticles are obtained with cubic structure, but in some cases, it is possible to obtain *Co* nanoparticles of a hexagonal structure [193]. It should be noted that in the case of the synthesis of cobalt nanoparticles by methods of “wet” chemistry under certain conditions, nanoparticles of a primitive cubic structure or ε-cobalt are formed [194]. The new *ε*-phase of cobalt was first observed namely in nanoparticles.

The hysteresis loops of a sample containing 5 wt. % *Co* in a polyethylene matrix were measured at 4.2K, 77K, and 295 K [138]. It has been found that unlike the samples containing *γ-Fe_2_O_3_* nanoparticles and considered above the system of nanoscale, magnetic *Co* particles in the sample are in a state of blocking both at low temperatures and at room temperature. Consequently, the blocking temperature for this sample is above room temperature. Upon cooling, the coercive force increases, reaching a value of 680 Oe at 4.2 K. Attention is drawn to the large magnitude of magnetization per atom. It was equal to 1.05 µ_B_/atom and 1.93 µ_B_/atom in the field of 4.5 kOe at 295 K and 4 kOe at 4.2 K, respectively, while it is known that the saturation magnetization of metallic Co at 4.2 K is equal to 1.7 µ_B_/atom. Thus, in the studied sample with *Co* nanoparticles in a polyethylene matrix the magnetic moment of cobalt is overestimated in comparison with the value for the bulk material. The possibility of an increase in the magnetic moment per atom in nanoparticles of 3d transition metals *Fe*, *Co*, and *Ni* was predicted earlier using theoretical calculations [195]. This an increase was observed experimentally in particles with a diameter of 10–25 Å in a polymer matrix, in 18–44 Å *Co* particles in micelles, and in experiments with beams of superparamagnetic nanoparticles of *Fe*, *Co*, and *Ni* [184,195]. In this case, the reason of the magnetic moment increase is probably laid in the fact that the core and shell of the particle have a different structure.

Along with the above, the effect of temperature exposure on the samples with *Co* nanoparticles was studied. The synthesized composite nanomaterials containing *Co* nanoparticles were exposed to temperature 280°C in air for 2 h. The results obtained on the change in magnetic properties after heating are presented in Table 8 [182].

As it can be seen from Table 8 the calcination does not lead to a change in the coercive force of the samples under study. This may be due to the oxidation resistance of cobalt-containing samples during heat treatment. At the same time, the magnetization value of the sample after calcination increased almost two times compared with the original sample. Most likely, this increase in magnetization may be due to the processes of crystal transformations in the structure of cobalt nanoparticles. It was also noted that cobalt-containing samples were more resistant to the effects of oxygen in the air when heated compared to iron-containing samples. Recall that when the latter were heated in air, it led to a decrease in their coercive force (*H_c_*) and magnetization (*M_R_*) (Table 8).

The investigations of cobalt nanoparticles allow us to better understand the specifics of the magnetic behavior of nanoscale objects. To date, it is clear that a simple model of single-domain, non-interacting nanoparticles in most cases turns out to be too rough although it describes many phenomena qualitatively correctly. Real nanoparticles are different in size, shape, internal structure, and therefore in properties. It is necessary to take into account the existence of a “core” and a “shell” in a particle with different magnetic characteristics as well as their interaction with each other. The magnetic anisotropy of cobalt nanoparticles is mainly determined by their surface. Thus, it depends on the properties of the matrix surrounding them.

Therefore, it is necessary to conclude that, now, there is no complete theory that would allow describing the magnetic properties of a set of nanoparticles. We can only talk about individual models that more or less successfully describe a limited number of experimental works. One of the reasons for this situation is the strong dependence of the properties of nanoparticles on the method of production and on the conditions of synthesis within the framework of one method, which makes the concept of “cobalt nanoparticle” insufficiently clearly defined.

**Figure 23:**
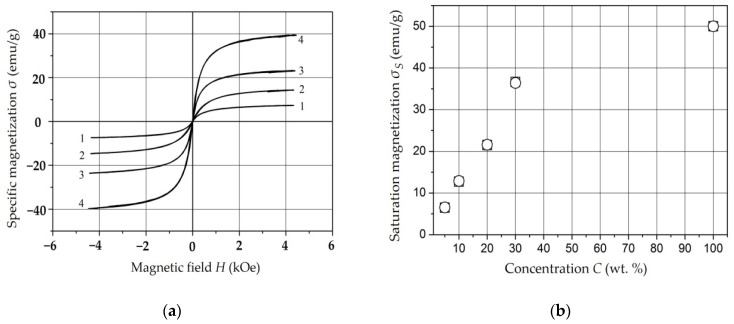
Demagnetization curves of the samples (*σ* (emu/g); *H* (kOe)) (**a**) and dependence of *σ_S_* (emu/g) on concentration of *NiFe_2_O_4_* nanoparticles (**b**) [151].

*Nanocomposites Containing Nickel Ferrite Nanoparticles.* The magnetization curves of the disk samples with a diameter of 5 mm and a thickness of 1.5 mm were carried out by means of an automated vibromagnetometer [151]. The demagnetization curves of the samples and the dependence of *σ_S_* (emu/g) on concentration *C* are shown in Figure 23.

It was found that the materials under study can be classified as soft magnets. The saturation magnetization *σ_S_* in them is achieved in fields of 2 kOe and increases with increase concentration *C*. Point *C* = 100 wt. % is corresponded to a tabular value. Thus, for a composite based on *NiFe_2_O_4_* nanoparticles stabilized in a HPPE matrix, the value of *σ_S_* = 38 is comparable to *σ_S_* = 50 for massive *NiFe_2_O_4_*.

### 4.6. Magnetic Properties of Composite Materials with Para- and Diamagnetic Nanoparticles

The magnetic characteristics of nanocomposites based on high-pressure polyethylene (HPPE) and magnetic nanoparticles (for example, iron- and cobalt-containing) have been studied in sufficient detail as shown above. At the same time, a wide range of non-magnetic composite nanomaterials has been obtained. These are materials based on *Mo*-, *Bi*-, *Pb*-, and *Re*-containing nanoparticles as well as materials based on *CdS*, *Cu*, *Hg*, and *CeO_2_* nanoparticles stabilized in a HPPE matrix [121,124,146,150,155,156,157,158,159,196,197]. In order to comprehensively study the properties of such composite nanomaterials, it is of interest to study their magnetic properties.

One of the most common methods of studying the magnetic characteristics of weakly magnetic substances is the Faraday method. The method is based on measuring the mechanical force acting on a sample in an inhomogeneous magnetic field. In this case, the sample under study is placed directly in the region of the maximum field gradient, and the sample size is chosen so that the field gradient does not change significantly in its volume [198].

**Figure 24:**
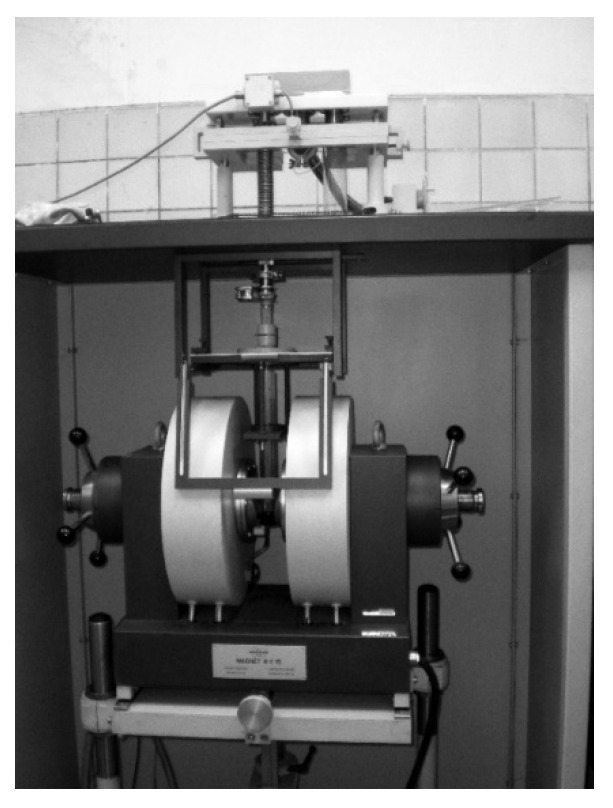
Photo of the experimental setup.

An experimental setup consisting of a Bruker magnetic field control system and Sartorius electronic microweights was used for measurements of the magnetic characteristics of weakly magnetic nanocomposites. The photo of this setup is presented in Figure 24. The main element of the magnetic field control system is a water-cooled electromagnet. To create an inhomogeneous magnetic field, the electromagnet has pole tips of a special shape that ensure the constancy (BdB/dz) of the magnitude in a sufficient working interval for measurements *Δz*. The maximum value of the magnetic field strength in the working area of the electromagnet gap is 6.5 kG.

Lever microbalance with electronic balancing and indication are used to measure the force acting on a sample placed in an inhomogeneous magnetic field. A microbalance has two measuring ranges with a sensitivity of 1 and 10 micrograms.

The disk samples with a diameter of 5 mm and a thickness of 1–2 mm were located at the bottom of the cup, so the condition of constancy of the magnetic field intensity gradient in the sample volume was met.

The force *F_z_* acting on the sample in an inhomogeneous magnetic field with a gradient dB/dz (in the CGS system):

(15) $$F_{z}=M\times \frac{dB}{dz}=V\times M_{J}\times \frac{dB}{dz}=V\times \chi \times \left(B\frac{dB}{dz}\right)=m\times \chi_{sp}\times \left(B\frac{dB}{dz}\right)$$

where *M* = *V* × *J_M_* is a magnetic moment in G × cm^3^, *V* is a volume in cm^3^, and *M_J_* is a magnetization of the sample in G; χ is a magnetic susceptibility of the sample; $\chi_{sp}$ = *χ*/*ρ* is a specific magnetic susceptibility of the sample in cm^3^/g; *m* is a sample weight in g; *F_z_* is a power in dynas; *B*d*B*/d*z* is in G^2^/cm.

It is necessary to know the force acting only on the sample for calculations. The measurements were made with an empty cup to this purpose, and the desired force was calculated as the difference:

*F_S_* = *F_T_* − *F_G_*, (16)

where indexes *S*, *T*, and *G* mean sample, total, and glass tube.

The product *B*d*B*/d*z* can be calculated according to the ratios (15) and (16) based on the results of measurements using a standard material with a known susceptibility. Copper sulfate *CuSO_4_·5H_2_O* was used as a standard material. Its magnetic susceptibility was assumed to be 5.94 × 10^−6^ cm^3^/g. The magnetic field strength in the working area of the electromagnet gap was measured by a teslameter as a function of the vertical coordinate and the field strength set by the electronic controller.

The dependence of the magnetization of samples on the magnetic field strength *J_sp_*(*B*) for weakly magnetic samples of nanocomposites can be obtained from the results of measurements of the force acting on the sample. The magnetic moment of the sample (in G × cm^3^) in the field of intensity *B*:

(17) $$M\left(B\right)=\frac{F_{z}}{\left(B\frac{dB}{dz}\right)}\times B$$

Then the specific magnetization of the sample *M_sp_* (emu/g):

(18) $$M_{sp}\left(B\right)=\frac{M\left(B\right)}{m}=\frac{F_{z}}{\left(B\frac{dB}{dz}\right)}\times \frac{B}{m}=\chi_{sp}\times B$$

**Figure 25:**
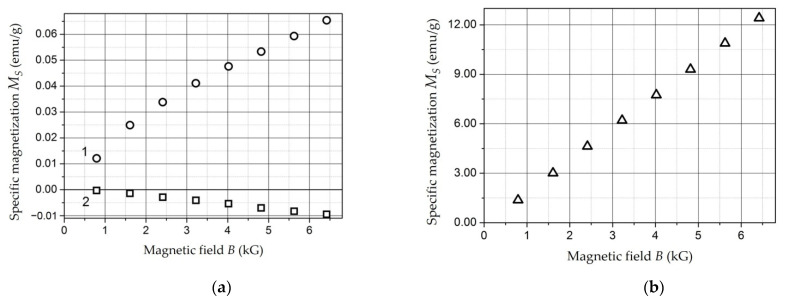
Magnetization curves of test samples: (**a**) *Al*, curve 1 (circles); *Zn*, curve 2 (squares); (**b**) *Ho_2_Ti_2_O_7_*.

The magnetization curves *M_sp_*(*B*) = *M_S_*(*B*) of the test samples are plotted based on the results of measurements and using the ratios (15)–(18). These dependencies are presented in Figure 25. The test samples were based on *Al*, *Zn*, and a sintered ceramic sample *Ho_2_Ti_2_O_7_*.

The specific magnetic susceptibility of the test samples was calculated using parameters *A_m_* and *C_m_* of the linear regression of the magnetization curve:

*M_S_*(*B*) = *A_m_*+*C_m_* × *B* (19)

The results obtained are presented in Table 9. It is correlated well with the literature data [199].

Then, according to the described method, measurements were performed, and magnetization curves were obtained for samples of weakly magnetic composite materials based on *Mo*, *Bi*, *CeO_2_*, *CdS*, *Pb*, *Cu*, *Hg*, and *Re* nanoparticles stabilized in a HPPE matrix.

First of all, it should be noted that the values of the specific magnetization of composite nanomaterials samples according to the research results did not exceed 0.1 emu/g in absolute values. This corresponds to the specific values for weakly magnetic substances including *Zn* and *Al*.

The studied samples can be attributed to two groups in accordance with the view of the obtained experimental dependences of magnetization on the magnetic field strength *M_S_*(*B*).

1. The samples based on *Mo*, *Bi*, *Hg*, and *Re* nanoparticles as well as a sample from unfilled HPPE that has undergone reaction treatment are characterized by negative magnetization at all values of the magnetic field strength. Its dependence *M_S_*(*B*) is satisfactorily described by a decreasing linear regression (19), while the value of the parameter *A_m_* of the linear regression can be assumed to be zero. Thus, such samples are diamagnets. The values of the specific magnetic susceptibility for these samples calculated by parameter *C_m_* of the linear regression of the magnetization curve are presented in Table 10.

As can be seen, the magnetic susceptibility of the composites based on *Bi*-, *Hg*-, and *Re* nanoparticles differs slightly from the susceptibility of unfilled HPPE. The embedding of *Mo*-containing nanoparticles into the matrix is lead to reduce $\chi_{sp}$ by almost two times. This may be due to the presence of paramagnetic molybdenum oxides of different valence in the composite based on *Mo*-containing nanoparticles.

**Figure 26:**
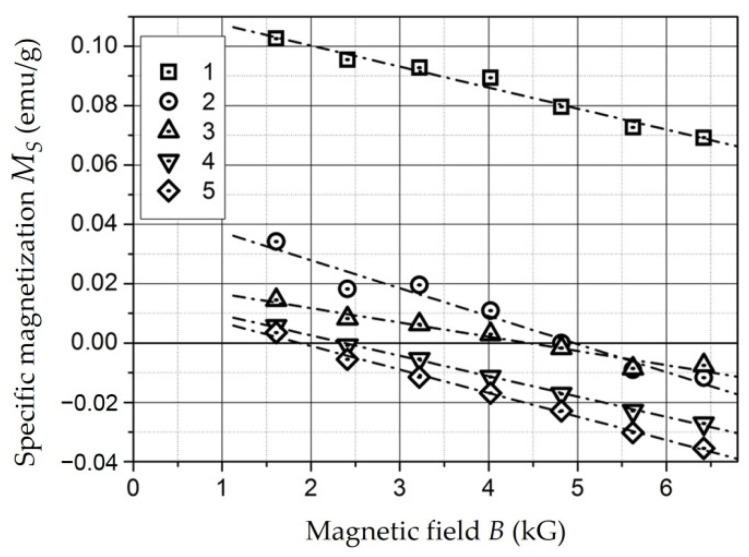
Magnetization curves of nanocomposites of the following compositions: (1) HPPE + 10% *Cu*; (2) HPPE + 10% *Pb*; (3) HPPE + 40% *CeO_2_*; (4) HPPE + 20% *CeO_2_*; (5) HPPE + 40% *CdS*.

2. The magnetization of the samples based on *Cu*-, *Pb*-, *CeO_2_*-, and *CdS*-containing nanoparticles stabilized in a HPPE matrix is changed from positive to negative at a certain value of the magnetic field strength. In this case, the dependence *M_S_*(*B*) at values of *B* = 1.5–6.5 kG is satisfactorily described by a decreasing linear regression of the form (19). The magnetization curves *M_S_*(*B*) of these samples are shown in Figure 26. The values of the specific magnetic susceptibility for these samples calculated by parameter *C_m_* of the linear regression of the magnetization curve are presented in Table 11 for fields with intensity *B* = 1.5–6.5 kG.

The behavior of the samples in the field B > 2 kG is diamagnetic. The vertical displacement of the magnetization curve is equal to the linear regression parameter *A*, and it makes sense for some “initial magnetization” of *M_S-0_*. It is achieved at *B* < 2 kG and remains constant with a further increase in the magnetic field strength.

The value *M_S-0_* for the studied samples is in the range of 0.014–0.12 emu/g. The source of this “initial magnetization” may be composite components with magnetic properties (para- and antiferromagnetic), the magnetization of which reaches saturation in weak fields. Their presence in low concentrations is due to both the manufacturing technology of nanocomposites based on HPPE containing nanoparticles and the complex phase composition of nanoparticles. For example, in the case of copper, copper (II) oxide *CuO* has antiferromagnetic ordering as well as copper and oxygen complexes with hydrocarbon molecules.

It should also be noted that the magnetic susceptibility of composites based on *Cu*-, *Pb*-, and *CdS*-containing nanoparticles differs slightly from the susceptibility of unfilled HPPE. The introduction of *CeO_2_* nanoparticles into the matrix leads to a decrease $\chi_{sp}$ at a relatively small value *M_S-0_*; i.e., as in the case of molybdenum, there is a paramagnetic contribution to the magnetization of the composite in fields with a strength of 1.5–6.5 kG. At the same time, there is a dependence of the effect on the concentration of the filler.

Thus, samples of nanocomposites based on *Cu*, *Pb*, *CeO_2_*, and *CdS* nanoparticles in fields up to 2 kG are magnetized in the direction of the magnetic field. This is demonstration of paramagnetic behavior. In the magnetic field strength range of 1.5–6.5 kG, its magnetization behavior is diamagnetic.

## 5. Properties of Composite Materials at Microwave Frequencies

The term microwave frequency range is sometimes used in a broad sense for the radio frequency range from 300 MHz to 300 GHz (wavelength range *λ* = 1–10^−3^ m). Electromagnetic oscillations in this region have common physical features and properties that distinguish them from other parts of the radio frequency spectrum. The design and technological implementation of measuring devices of these ranges also has general principles. The measurement methods based on measuring lines, resonant and quasi-optical methods, as well as their combinations are used to study the properties of materials in the microwave range. These methods make it possible to obtain information on the complex dielectric and magnetic permeability, surface impedance, coefficients of reflection, transmission, and absorption of microwave radiation power as well as to carry out field tests of various types of functional coatings based on nanocomposites in the microwave range [200].

**Figure 27:**
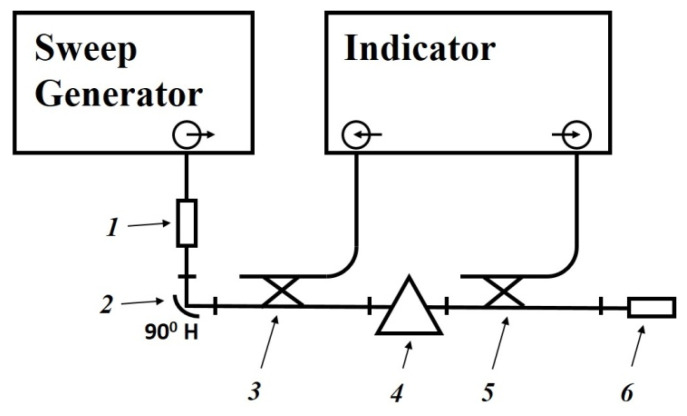
The scheme of the setup for measuring attenuation. (1) attenuator, (2) adapter, (3) incident wave DC, (4) measured object, (5) transmitted wave DC, and (6) matched load.

**Figure 28:**
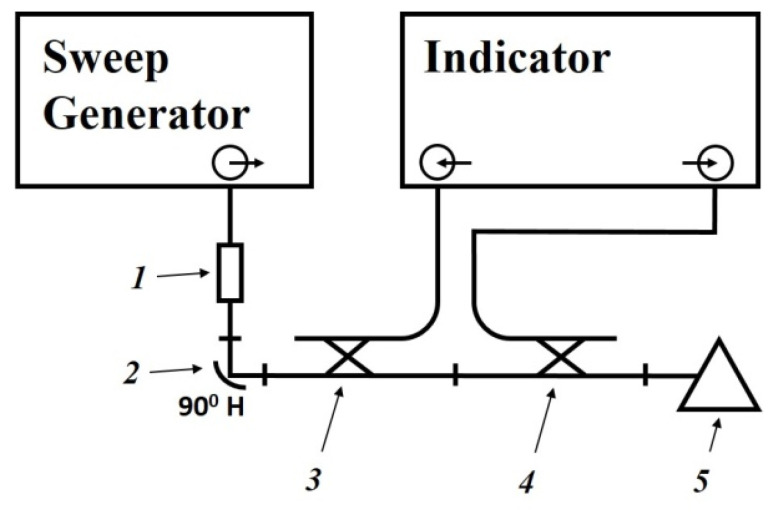
The scheme of the setup for measuring standing wave ratio: (1) attenuator, (2) adapter, (3) incident wave DC, (4) reflected wave DC, and (5) measured object (reference load).

A measuring set up for investigation of the possibility of using the obtained composite materials for the tasks of electromagnetic compatibility, noise protection, radio masking, and protection of biological objects from electromagnetic radiation was assembled [21]. This set up was used to study the properties of composite materials based on metal-containing nanoparticles in an HPPE matrix by a quasi-optical method at a frequency of 30 GHz using a standing wave coefficient measuring set (KSVN R2-65, Russia) with a cross section of the waveguide of 7.2 × 3.4 mm^2^. The schemes of the setups for measuring standing wave ratio (SWR) and attenuation are shown in Figure 27 and Figure 28, respectively.

The method is based on measuring the coefficients of reflection and attenuation of the power of an electromagnetic wave in the path containing the test sample using directional couplers. The attenuation coefficient (*A*) is calculated from the known values of incident power (*P_inc_*) and transmitted power (*P_trans_*)according to the formulae

*A*= −10 × log(*P_trans_*/*P_inc_*), (dB) (20)

and standing wave ratio *K_swU_* = (1 + |*r*|)/(1 − |*r*|), where $r=\sqrt{P_{refl}/P_{inc}}$, *P_refl_*, and *R* = *r*^2^ = *P_refl_*/*P_inc_* are the voltage reflection coefficient, reflection power, and power reflection coefficient, respectively.

The sample is placed in a segment of the waveguide of the corresponding section. Based on measurements of attenuation and reflection for an empty and filled waveguide, the reflection R and loss L coefficients for the material under study are calculated.

**Figure 29:**
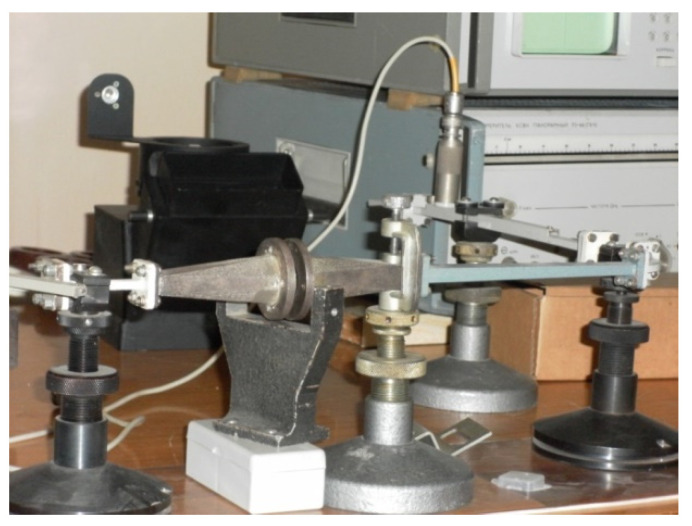
Photograph of a measuring stand with a horn measuring cell.

A system of two matched emitting horns connected to the waveguide of an SWR meter, the so-called quasi-optical technique [201,202], can be used as a measuring cell (Figure 29). It is necessary to have the samples under study with sufficient sizes. The sample in this case is placed between the receiving and transmitting horns.

**Figure 30:**
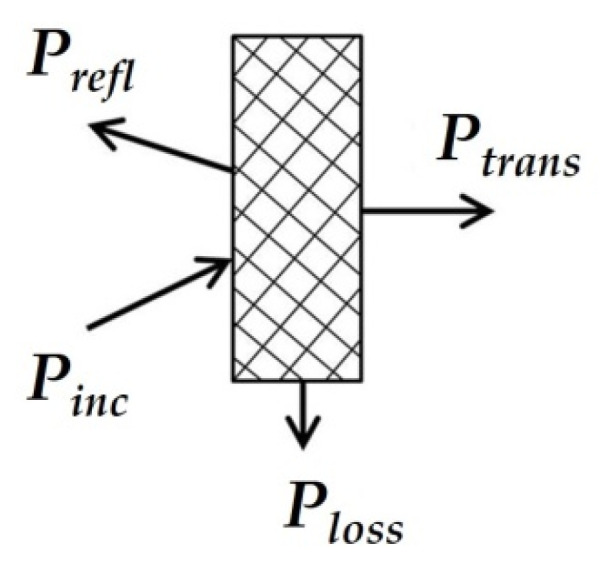
Scheme of power balance in the interaction of EMR with the sample.

The measurements were carried out at a fixed frequency of 30 GHz using a horn measuring cell. The samples for measurements were discs of 15–30 mm in diameter and of 1–3 mm thick. The total reflection level was calibrated using a short circuit at the output of an empty measuring cell. The photograph of a microwave set up with a horn measuring cell and the scheme of power balance in the interaction of EMR with the sample are presented in Figure 29 and Figure 30, respectively.

The specific radio absorption *a*(dB/cm) is the relation the attenuation coefficient *A* to the sample thickness. It allows compare the efficiency of microwave signal shielding by samples characterized by different thicknesses.

The reflection coefficient (R*) was measured by the ratio of the reflected microwave power to the incident power:

(21) $$R^{*}=10\times \log\frac{P_{refl}}{P_{inc}},\;(\mathrm{dB})$$

The reflection coefficient *R* in relative units was calculated by (22):

(22) $$R=\frac{P_{refl}}{P_{inc}}$$

The relative loss coefficient *L* was calculated from the known values *R* and *A* according to the relationship:

(23) $$P_{inc}=P_{trans}+P_{refl}+P_{loss}$$

(24) $$L=\frac{P_{loss}}{P_{inc}}$$

The results of measurements of the coefficients of reflection, radio absorption, attenuation, and losses for samples of composite materials based on metal-containing nanoparticles stabilized in an HPPE matrix are presented below.

### 5.1. Iron-Containing Composites

**Figure 31:**
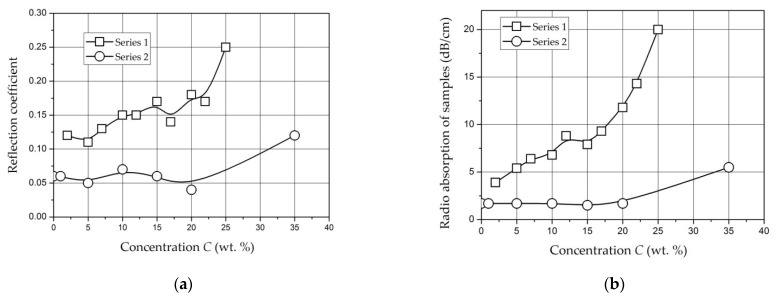
Dependence of (**a**) the reflection coefficients and (**b**) the specific radio absorption of samples on the mass concentration of iron in the composite.

Studies were carried out on two series of composite materials based on iron-containing nanoparticles synthesized from iron pentacarbonyl *Fe(CO)_5_* with a metal-containing component concentration of 0–35 wt. %. The results of measuring the reflection coefficients and the radio absorption of samples at a frequency of 30 GHz by the quasi-optical method are shown in Figure 31.

According to Mössbauer spectroscopy data, the samples of the two series have differences in the composition of nanoparticles:

- Mass fraction of α-iron (metal core) in the composition of nanoparticles for series 1 is 20% (the size of the core is about 10 nm, and it is clearly expressed); for series 2, it is less than 5% (the metal core is very weakly expressed).
- The rest of the iron is included in the composition of nanoparticles in the form of *Fe_2_O_3_* and *Fe_3_O_4_* oxides in approximately equal proportions. Both oxides form an amorphous shell around the metal core (there is no “massive” oxide phase).

The higher content of the oxide phase in the samples of series 2 is associated with the oxidation of metallic iron reduced in the course of the thermal decomposition reaction of the precursor. The inert atmosphere (purging with argon) created in the reactor contributes to a decrease in the oxidation rate and the preservation of a massive metal core.

Comparison of the specific radio absorption and reflection coefficients at a frequency of 30 GHz for the two series makes it possible to reveal the difference in the mechanisms of EMW power absorption in the material. The specific radio absorption and reflection coefficients for samples from series 2 (with a weakly pronounced metal core) differ slightly from the values for unfilled HPPE.

A polymer containing “core-shell” nanoparticles with a metal core of α-iron (series 1) is characterized by a monotonic increase in the specific radio absorption, which indicates the presence of additional (to an unfilled matrix) absorption mechanisms. Such mechanisms are magnetization reversal losses and ring currents in the metal core of nanoparticles [203,204].

The coefficients of attenuation, reflection, and loss at frequencies of 25 and 30 GHz for samples based on iron-containing nanoparticles synthesized from iron formate (*Fe(HCOO)_3_*) are presented in Table 12.

An increase in the absorptive capacity of the samples with an increase in *C_Fe_* is caused by a decrease in the specific volume resistance due to Joule losses and the presence of a magnetic phase in the composite due to losses to magnetization reversal [173].

### 5.2. Cobalt-Containing Composites

It was found that a cobalt-containing composite material with *C_Co_* = wt. 30% synthesized from *Co(HCOO)_2_* has the most optimal ratio of *A* and *R* among the studied samples of polymer composite materials based on metal-containing nanoparticles (Table 13).

The difference in the attenuation and reflection characteristics of *Co*-02 and *Co*-03 samples can be explained by different pressing temperatures: 230 °C and 280 °C, respectively. Obviously, a higher pressing temperature made it possible in this case to obtain a denser block sample (tablet), in which there are no cavities filled with air. This was established by simple visual inspection of the cross section of the tablet. It was found that the *Co*-03 sample has the maximum attenuation coefficient *A*.

**Figure 32:**
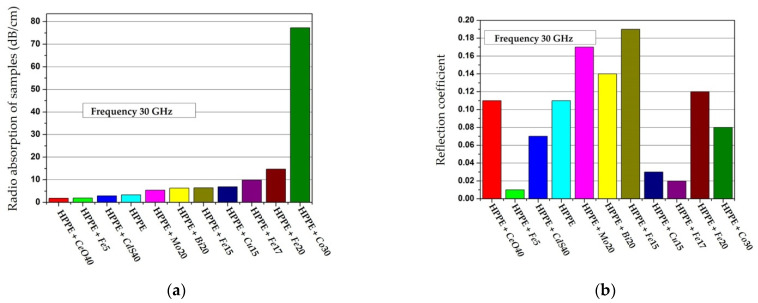
(**a**) Radio absorption and (**b**) reflection coefficients of composite materials based on metal-containing nanoparticles.

The specific radio absorption and reflection coefficients of various samples of filled polymer composites are presented in Figure 32 measured at frequency of 30 GHz [205]. Obviously, the values of the specific radio absorption and reflection coefficients are determined by the composition of the samples.

**Figure 33:**
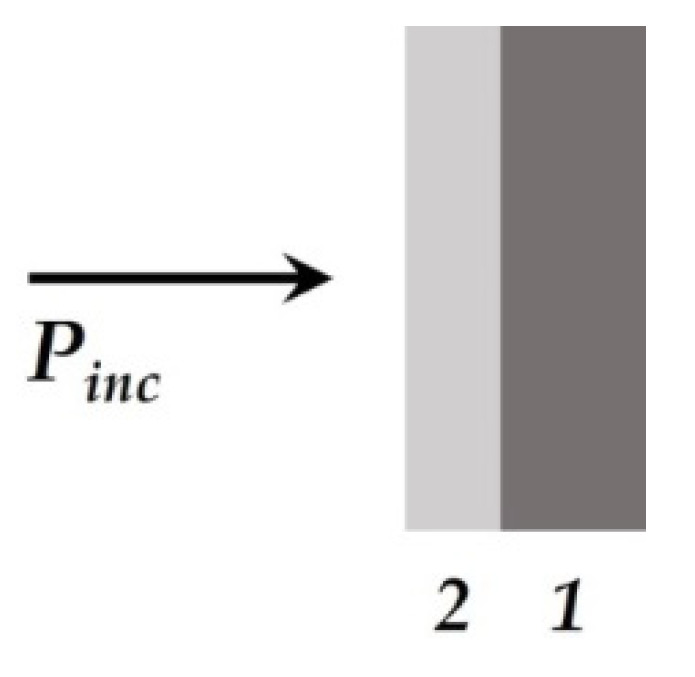
Scheme of the formation of a two-layer combination of samples: (1) base sample Co-03 and (2) additional sample.

In addition, the attenuation and reflection characteristics of two-layer combinations of samples of composite materials based on metal-containing nanoparticles at a frequency of 30 GHz were studied. The test objects were a two-layer combination of tablets. Base sample 1 in all combinations is a tablet of a composite based on cobalt-containing nanoparticles with *C_Co_* = wt. 30% synthesized from *Co(HCOO)_2_* (sample *Co*-03). Over this sample, another of composition 2 was superimposed from the side of the generator (Figure 33).

**Figure 34:**
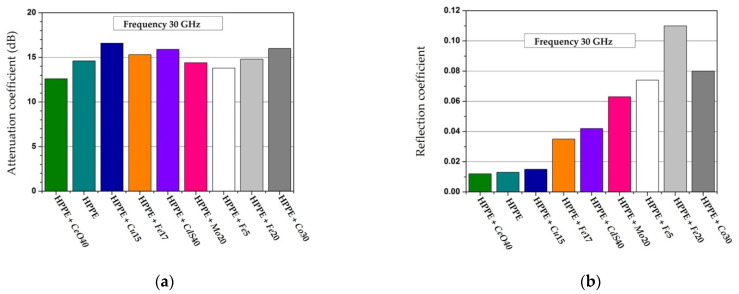
(**a**) Attenuation coefficient and (**b**) reflection coefficients of two-layer combinations of samples of composite materials based on metal-containing nanoparticles.

The choice of the sample Co-03 as the base 1 was due to the fact that this sample had the highest attenuation value according to the results of individual measurements. The results of studies of the attenuation and reflection characteristics of two-layer combinations are summarized in Figure 34. The rightmost column in the diagrams corresponds to the attenuation and reflection coefficients of a single sample Co-03. The remaining columns give the values of the attenuation and reflection coefficients of two-layer combinations with an additional sample, the composition of which is indicated in the diagram. The total thickness of combinations during measurements was 3.2–5.5 mm.

From the data presented in Figure 34, we can conclude that the attenuation of two-layer combinations is determined by the base (absorbing) layer, and the reflection is determined by the layer facing the generator (matching, radio transparent) at a frequency of 30 GHz.

### 5.3. Molybdenum-Containing Composites

The measured values of the coefficients of attenuation and reflection for samples of composite materials with different concentrations of molybdenum at a frequency of 30 GHz are presented in Table 14.

A slight increase in the attenuation coefficient with an increase in the mass concentration of the filler is associated with additional losses due to the polarization of nanoparticles. This also explains the insignificant increase in the reflection coefficient since with an increase in the permittivity for nonmagnetic media, the wave resistance of the material decreases [146].

### 5.4. Lead-Containing Composites

The measured values of the specific radio absorption ***a*** and reflection R coefficient at a frequency of 30 GHz for samples of composite materials with different concentrations of lead are presented in Table 15.

A comparative analysis of the specific radio absorption of the filled and unfilled composites under study has shown that the filler of electrically conductive lead nanoparticles does not lead to a significant increase in the absorption of electromagnetic radiation. Thus, the main mechanism of electromagnetic wave power absorption in the microwave range for the studied composites is ion relaxation [150].

### 5.5. Composites with Nickel Ferrite Nanoparticles

**Figure 35:**
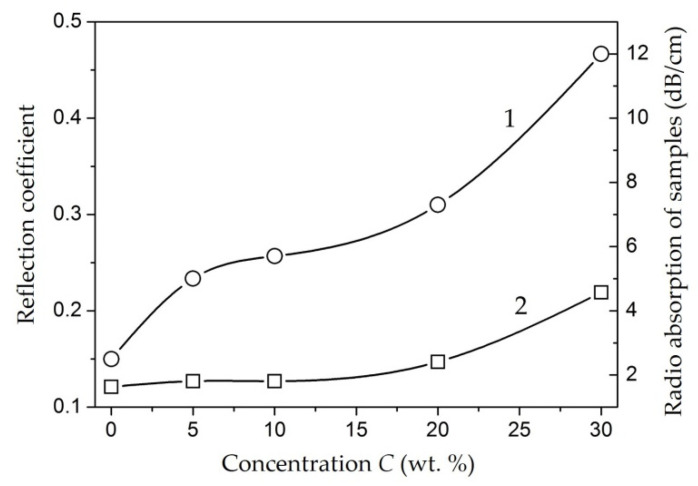
Dependence of specific radio absorption (1) and reflection coefficient (2) on concentration of nickel ferrite nanoparticles [151].

The tablet samples 1.5 mm thick were studied. As the concentration increases, R and α increase (Figure 35). In this case up to *C* = 20 wt. % the values of the specific radio absorption α lie in the range of 5–7 dB/cm with a relatively low *R* equal to 0.13–0.15. These values are not much higher than for unfilled HPPE 2.5 dB/cm and 0.12, respectively. The main increase in the values of *α* = 12 dB/cm and *R* = 0.22 is observed in the concentration range of 20–30 wt. % [151]. The increase in the absorbing capacity of the samples with increasing *C*, as in the case of iron-containing nanoparticles, is caused by a decrease in the specific volume resistance due to joule losses and the presence of a magnetic phase in the composite due to losses to magnetization reversal.

### 5.6. Rhenium-Containing Composites

The measured values of the specific radio absorption and reflection coefficients for samples of rhenium-containing composite materials synthesized under various conditions at a frequency of 30 GHz are presented in Table 16.

A higher content of rhenium compounds with a low degree of oxidation (*Re*, *ReO_2_*, *ReO_3_*) leads to a higher mobility of electron shells and polarizability of nanoparticles as well as to an increase in dielectric losses at microwave frequencies. This results in higher values of *a* for samples *Re*-01 and *Re*-04. Conversely, the predominance of compounds with a high degree of oxidation (*Re_2_O_7_*) does not make a noticeable additional contribution to the polarization of the composite and dielectric losses. Therefore, the values of *a* for the *Re*-02 sample slightly differ from those for unfilled polyethylene [124,155].

In conclusion, the summary results of the study of the coefficients of attenuation *A*, reflection *R*, and losses *L* for various metal-containing nanoparticles in HPPE matrix materials are presented in Table 17.

The analyses of data have shown that the coefficients of attenuation *A*, reflection *R*, and loss *L* of the samples at a frequency of 30 GHz lie in the range of 0.3–16, 0.03–0.53, and 0–0.9 dB, respectively, with sample thicknesses of 1–3 mm. The values of the coefficients of attenuation *A*, reflection *R*, and loss *L* are determined by the composition and thickness of the samples.

It can be seen from the data presented that polymer composites based on metal-containing nanoparticles of various compositions possess significantly different characteristics of absorption and reflection of the power of an electromagnetic wave at a frequency of 30 GHz. In this case, samples of composite materials containing magnetic nanoparticles of cobalt, iron, and nickel ferrite characterized by the highest attenuation coefficient. Based on such materials, effective shielding and radio-absorbing elements of radio engineering devices can be created. Two-layer combinations of materials with nanoparticles of different compositions manufactured using the same technology can provide materials with the required attenuation and reflection characteristics. This shows the possibility of creating radio-absorbing, radio-transparent, shielding and matching materials with desired properties.

## 6. Multilayer Coatings with Variable Electrodynamics Characteristics Based on Filled Polymer Matrices

The problem of reducing EMR reflections from cladding materials is solved when designing anechoic chambers. Pyramids of polyurethane foam impregnated with colloidal graphite suspensions or polymer pyramids filled with samples of electrically conductive papers are often used as facing materials of anechoic chambers [24,205].

Coatings that are impedance-matched to the medium can be created on the basis of multilayer structures in which the wave impedance decreases as one immerses in it. This makes it possible to avoid sudden jumps in wave resistance and, consequently, unwanted reflections [206]. The simplest variant of such a structure is a two-layer coating of a material with different conductivity. In this case, the upper layer with lower values of *σ* is often called a matching layer, and the lower one with large k values of *σ* is called an absorbing one. The absorption coefficient for multilayer coatings depends in a rather complex way on the dielectric and magnetic properties as well as the thickness of each layer. It can be calculated, for example, through the product of the wave transmission matrices of each layer and the subsequent reduction of the components of the resulting total transmission matrix to the reflection coefficients. Mathematical modeling of the frequency characteristics of the reflection coefficients was carried out using a model of multilayer coatings to solve the problem of optimal choice of the parameters of the RAM. Structured polymer-based materials are widely used in the development of new structural composite nanomaterials [207,208,209,210,211,212,213,214,215,216].

### 6.1. Mathematical Modeling of the Interaction of a Multilayer Coating with Electromagnetic Radiation

The results of the interaction of the developed structure with EMR can be different. It depends on the radiation intensity, the number and thickness of the layers, and their electrical conductivity and electrophysical characteristics. The process of EMR interaction with the coating is characterized by the reflected (*R*), absorbed (*Q*), and transmitted energy (*T*). It is known that the values of *R*, *Q*, and *T* are determined by the complex values of the dielectric, magnetic permeability of samples, the wavelength of radiation, and the thickness of the sample in a simple idealized model of the material. Within the framework of a theoretical model with complex values of dielectric permittivity (magnetic permeability is equal to 1), the dependences of changes in values *R*, *Q*, and *T* are studied by the interaction of electrically conductive materials of various conductivity with electromagnetic waves of *λ* = 0.1, 1.0, and 10 cm.

The problem of the passage of EMR through a multilayer coating is considered. Each layer was characterized by the parameters of complex permittivity, conductivity, and thickness. The process of interaction of EMR with electrically conductive samples of composite materials is characterized by reflected, absorbed, and transmitted energies.

The equations for determination of the coefficients of reflection and transmission through a system of five layers have the following form [217]:

(25) $$\begin{array}{l} E_{1}^{+}+E_{1}^{-}=E_{2}^{+}+E_{2}^{-}\\ E_{1}^{+}-E_{1}^{-}=Z_{12}\times \left(E_{2}^{+}-E_{2}^{-}\right)\\ E_{2}^{+}e^{ik_{2}d_{2}}+E_{2}^{-}e^{-ik_{2}d_{2}}=\left(E_{3}^{+}e^{ik_{3}d_{2}}+E_{3}^{-}e^{-ik_{3}d_{2}}\right)\\ E_{2}^{+}e^{ik_{2}d_{2}}-E_{2}^{-}e^{-ik_{2}d_{2}}=Z_{23}\times \left(E_{3}^{+}e^{ik_{3}d_{2}}-E_{3}^{-}e^{-ik_{3}d_{2}}\right)\\ E_{3}^{+}e^{ik_{3}d_{23}}+E_{3}^{-}e^{-ik_{3}d_{23}}=\left(E_{4}^{+}e^{ik_{4}d_{23}}+E_{4}^{-}e^{-ik_{4}d_{23}}\right)\\ E_{3}^{+}e^{ik_{3}d_{23}}-E_{3}^{-}e^{-ik_{3}d_{23}}=Z_{34}\times \left(E_{4}^{+}e^{ik_{4}d_{23}}-E_{4}^{-}e^{-ik_{4}d_{23}}\right)\\ E_{4}^{+}e^{ik_{4}d_{234}}+E_{4}^{-}e^{-ik_{4}d_{234}}=\left(E_{5}^{+}e^{ik_{5}d_{234}}+E_{5}^{-}e^{-ik_{5}d_{234}}\right)\\ E_{4}^{+}e^{ik_{4}d_{234}}-E_{4}^{-}e^{-ik_{4}d_{234}}=Z_{45}\times \left(E_{5}^{+}e^{ik_{5}d_{234}}-E_{5}^{-}e^{-ik_{5}d_{234}}\right)\\ E_{5}^{+}e^{ik_{5}d_{2345}}+E_{5}^{-}e^{-ik_{5}d_{2345}}=\left(E_{6}^{+}e^{ik_{6}d_{2345}}+E_{6}^{-}e^{-ik_{6}d_{2345}}\right)\\ E_{5}^{+}e^{ik_{5}d_{2345}}-E_{5}^{-}e^{-ik_{5}d_{2345}}=Z_{56}\times \left(E_{6}^{+}e^{ik_{6}d_{2345}}-E_{6}^{-}e^{-ik_{6}d_{2345}}\right)\\ E_{6}^{+}e^{ik_{6}d_{23456}}+E_{6}^{-}e^{-ik_{6}d_{23456}}=E_{7}^{+}e^{ik_{7}d_{23456}}\\ E_{6}^{+}e^{ik_{6}d_{23456}}-E_{6}^{-}e^{-ik_{6}d_{23456}}=Z_{67}\times E_{7}^{+}e^{ik_{7}d_{23456}} \end{array}$$

Here, *E_j_*, *d_a_*, *d_ab_*… = *d_a_*+ *d_b_*+…, and *k_a_* = *α_a_*+*iβ_a_* are the field in the *j*-th layer, thickness of the *a*-th layer, thickness of the combined layer, and complex wave vector, respectively.

(26) $$Z_{ab}=\frac{\mu_{a}k_{b}}{\mu_{b}k_{a}}$$

(27) $$\begin{array}{l} \alpha_{a}=\omega \times {\left(\frac{\mu_{a}\varepsilon_{a}}{2}\times \left(\sqrt{1+\frac{\sigma_{a}^{2}}{\varepsilon_{a}^{2}\omega^{2}}}+1\right)\right)}^{1/2}\\ \beta_{a}=\omega \times {\left(\frac{\mu_{a}\varepsilon_{a}}{2}\times \left(\sqrt{1+\frac{\sigma_{a}^{2}}{\varepsilon_{a}^{2}\omega^{2}}}-1\right)\right)}^{1/2} \end{array}$$

*ω*, *ε_a_*, and *μ_a_* are the cyclic frequency and dielectric and magnetic permeability of the *a*-th layer.

Reflection and transmission coefficients of the electromagnetic wave:

(28) $$R={\left|\frac{E_{1}^{-}}{E_{1}^{+}}\right|}^{2},\;\;\;\;T={\left|\frac{E_{7}^{+}}{E_{1}^{+}}\right|}^{2}\;\;$$

Part of absorbed energy:

(29) Q=1−R−T

Calculations were performed for combinations of composite layers of different electrical conductivity and thickness located on metal and dielectric substrates.

**Figure 36:**
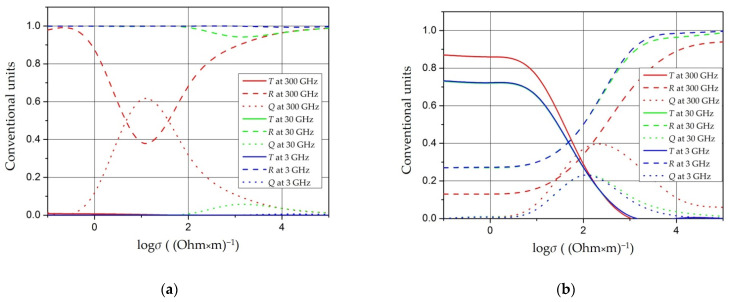
The dependences of the reflection *R*, transmission *T*, and absorption *Q* coefficients on the electrical conductivity of the composite layer on (**a**) metal substrate with electrical conductivity *σ_m_* = 10^7^ (Ohm × m)^−1^ and (**b**) ceramic substrate with *ε_c_* = 10 at the different incident radiation frequencies of 300 GHz (dashed line), 30 GHz (solid line), and 3 GHz (dotted line).

The dependences of the reflection *R*, transmission *T*, and absorption *Q* coefficients on the electrical conductivity of the composite layer on the substrate at the different incident radiation frequencies of 300 GHz (dashed line), 30 GHz (solid line), and 3 GHz (dotted line) are shown in Figure 36. A metal substrate with electrical conductivity *σ_m_* = 10^7^ (Ohm × m)^−1^ (Figure 36a) and ceramic substrate with *ε_c_* = 10 (Figure 36b) are used as substrates for composite layer. The thickness of the composite layer, the dielectric permittivity *ε*, and the magnetic permeability *μ* of materials are 0.07, 5, and 1 mm, respectively.

**Figure 37:**
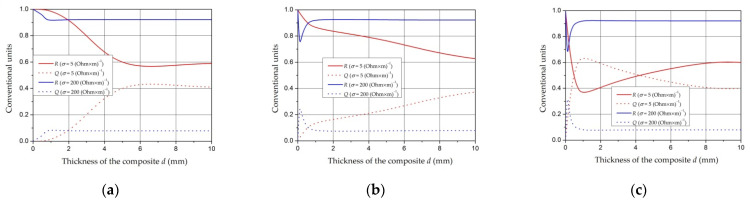
The dependencies of the reflection coefficients R (solid line) and absorption coefficients *Q* (dotted line) on the composite layer thickness *d*. Composite layer on a *Cu* substrate (**a**) without an intermediate layer, (**b**) with dielectric (*ε* = 50) intermediate layer with a thickness of 5 mm, and (**c**) with dielectric (*ε* = 50) intermediate layer with a thickness of 10 mm.

The dependences of the reflection coefficients *R* (solid line) and absorption coefficients *Q* (dotted line) on the composite layer thickness *d* are presented in Figure 37. The composite layers with *σ* = 5 (Ohm × m)^−1^ (solid line) and *σ* = 200 (Ohm × m)^−1^ (dotted line) were placed on a *Cu* substrate without (a) or with intermediate dielectric (*ε* = 50) layer with thicknesses (b) 5 mm or (c) 10 mm.

Thus, within the framework of the two-layer coating model, the difference in the effects of the interaction of EMR with conducting samples located on a dielectric and metal substrate is demonstrated. It is shown that in order to achieve optimal (maximum) levels of reflection and absorption, it is necessary to fit the appropriate values of *σ*, *d*, and *ε* of the layers at the radiation frequency changes.

### 6.2. Experimental Study of Multilayer Coatings with Variable Electrodynamic Characteristics

Polyvinyl chloride (PVC) plastisols are a dispersion of polyvinyl chloride and/or copolymer in liquid plasticizers. The amount of plasticizers (dibutylphthalates, dialkyl phthalates, etc.) ranges from 30 to 80%. Plastisols are converted into highly bonded masses at heating to 170 °C. This occurs as a result of the acceleration of the swelling process (gelatinization).After cooling, these materials become elastic. Fine powders of magnetically soft, dielectric and conductive materials can be introduced into the PVC plastisol matrix [218,219,220].

The samples of composite materials based on polyvinyl chloride plastic with dioctylphthalate plasticizer were made [221]. Filler was added to the plastic mixture in a given mass concentration and thoroughly mixed. Then the resulting mass was filled into a mold and cured in a drying cabinet at a temperature of 160–180 °C. Thermally expanded graphite (TEG), annealed barium titanate powder (BaTiO_3_), colloidal graphite (CG), and cobalt-based amorphous magnetic alloy powder (AMAG) were used as a filler. As a result, a set of plane-parallel samples with a diameter of 40 mm and a thickness of 1.4–4.3 mm with a different composition and concentration of filler as well as samples of unfilled polymer were produced [222,223].

The electrically conductive properties of the samples at direct current as well as the dielectric constant and the tangent of the loss angle at frequencies up to 1 MHz were investigated. The measurements were carried out using the Agilent 4339B high-resistance meter and the Agilent E4980A LCR meter with measuring cells included in the instrument set. The results of measurements of electrophysical properties are presented in Table 18.

**Figure 38:**
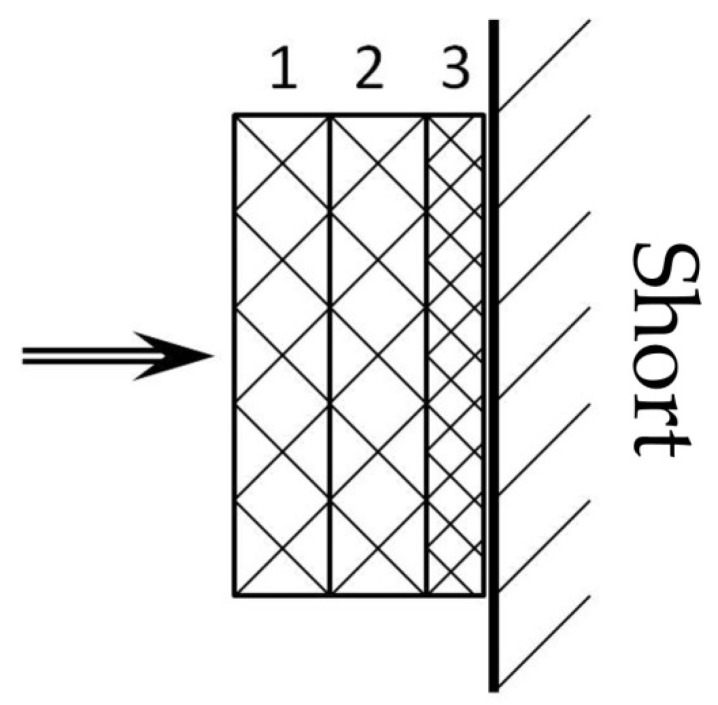
Scheme of a three-layer composite coating in a short-circuited path. (1) and (2) are the absorbing composite layer; (3) is the underlying dielectric layer. The arrow shows the direction of an incident wave.

From the set of the materials obtained, it was necessary to select appropriate composites for an absorbing gradient layer. Such a gradient layer consists of layers 1 and 2 (Figure 38). In addition, it was necessary to choose a composite for the underlying dielectric layer 3 with relatively low losses and high permittivity. Based on these materials, it was possible to form appropriate multilayer coatings.

Based on the results of low-frequency measurements as well as measurements of reflection coefficients *R* and absorption *Q* at a frequency of 30 GHz presented in Table 18, the combinations of three layers were selected for the study. These combinations are presented Table 19.

**Figure 39:**
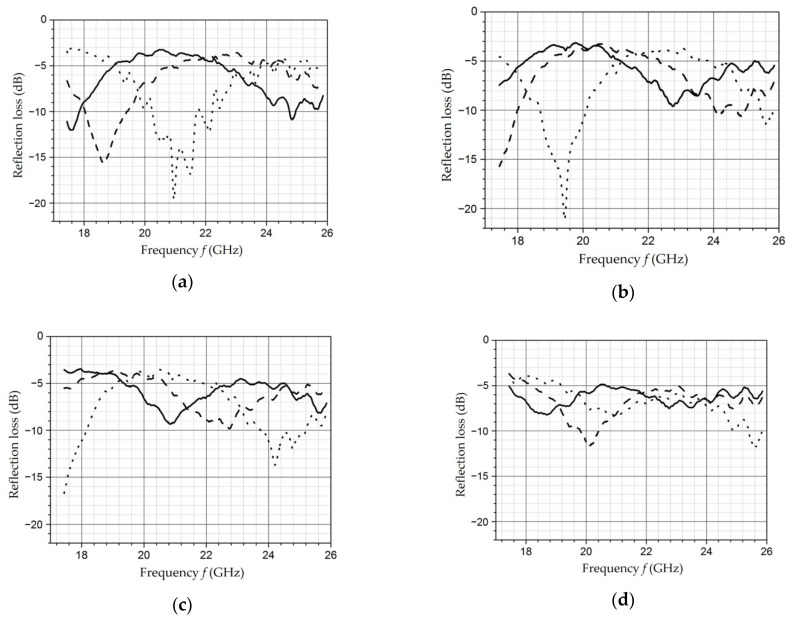
The reflection losses of various variants of three-layer coatings. As for the underlying dielectric layer with *ε* ≈ 4–10, the composition of the filler was varied, such as (**a**) no filler, (**b**) *BaTiO_3_*, (**c**) *BaTiO_3_* + CG, and (**d**) *BaTiO_3_* + AMAG.

The dependences of the reflection coefficient (reflection loss) on the frequency for three-layer coatings based on PVC matrix in the frequency band of 17.44–25.86 GHz obtained by means a measuring stand based on a vector circuit analyzer are presented in Figure 39. The investigated coating options consisted of two layers of an absorbing composite with different mass concentrations of TEG filler 2% and 3% (solid line), 1% and 3% (dashed line), and 1% and 2% (dotted line). As for the underlying dielectric layer with *ε* ≈ 4–10, the composition of the filler was varied, such as (a) no filler, (b) *BaTiO_3_*, (c) *BaTiO_3_* + CG, and (d) *BaTiO_3_* + AMAG.

Thus, it was shown that, on the basis of filled polymers, it is possible to create effective radio-absorbing coatings with a gradient distribution of electrically conductive, dielectric, and magnetic fillers in the polymer matrix. The developed technology based on PVC with fillers of various types makes it possible to obtain composite materials and multilayer coatings based on them with variable electrodynamic characteristics. This conclusion is confirmed by studies of the electro-physical properties of these materials at various frequencies.

Therefore, multilayer electrodynamic structures containing combinations of layers of filled polymer composite materials with nanoparticles of different compositions and manufactured using a single technology will make it possible to create electrodynamic media and coatings with the required electro-physical characteristics (absorption, transmission, and reflection). Within the framework of models of two-layer and three-layer coatings, the difference in the effects of the interaction of electromagnetic radiation with conductive layers located on a dielectric and metal substrate is demonstrated. It is shown that in order to achieve optimal (maximum) values of reflection and absorption of electromagnetic radiation in the appropriate frequency range, it is necessary to fit the required values of the layer thickness, specific conductivity, and permittivity. Such approach allows designers to create new effective shielding materials that can effectively vary the shielding, absorbing, and matching characteristics of coatings over a wide frequency band.

## 7. Conclusions

The rapid growth of digitalization and the widespread use of microprocessor technology in such areas as intelligent electronics, electrical engineering, telecommunications, consumer electronics, transport, and medicine complicate the solution of the problem of electromagnetic compatibility due to crosstalk. Electromagnetic waves from various natural and technical electrical sources are radiated into the atmosphere and create an electromagnetic background in the environment with different frequencies and intensities. Such electromagnetic interference can interrupt the operation of other devices and endanger human health. Thus, all electronic devices, regardless of their size and purpose, require advanced electromagnetic shielding materials and methods that can limit or completely block the transmission of electromagnetic waves in the atmosphere.

The shielding material must possess small weight, high conductivity, and high permittivity; be economical, easy to process, and flexible; and have a wide operating frequency range depending on the application. This is due to the growing demand for modern electronics. At present, not only innovative radio-absorbing materials are needed for shielding and suppressing excessive electromagnetic interference but also complex composite coatings with programmable electrical properties.

An analysis of the current state-of-the-art on the development of composite materials based on filled polymers, including radio-absorbing and radio-transparent materials, as well as electromagnetic shields designed to ensure electromagnetic compatibility, allows us to draw the next conclusions:

1. Most promising materials are based on nanosized fillers, including those possessing magneto-dielectric properties.
2. Evaluation of the effectiveness of the material is based on the analysis of the following parameters: reflection and attenuation coefficients in terms of power, the width of the operating frequency range, the values of dielectric and magnetic permeability and losses, as well as weight and size.
3. Materials that allow complex technological processing, for example, multilayer gradient compositions, have an advantage.
4. High-density polyethylene is most suitable as a stabilizer matrix. The technology of its production and processing is well-developed. It has excellent electrical properties, process ability, and chemical inertness in most environments and is widely used in electronic products.

The paper presents a comprehensive and methodical review of existing modern polymer composites for radio shielding. A wide class of modern filled polymeric materials with dielectric and magneto-dielectric losses is considered. These materials make it possible to create effective radio-absorbing materials that provide a low level of reflection coefficient in the microwave range.

The main methods of creating broadband RAMs are considered. One of them is the use of materials with special dispersion laws of dielectric and magnetic permeability. RAMs developed on this principle have already found wide application in reducing the radar visibility of various objects.

It is shown that the polymer composites based on “core-shell” nanoparticles, depending on their composition, have significantly different characteristics of absorption and reflection of electromagnetic wave power. The conditions of the technological process make it possible to control the composition of nanoparticles. This leads to the possibility of creating radio-absorbing materials with desired properties. Multilayer electrodynamic media containing combinations of layers of filled polymer composite materials with nanoparticles of different compositions and manufactured using a single technology will make it possible to create electrodynamic media and coatings with the required electro-physical characteristics of absorption, transmission, and reflection.

Within the framework of the two-layer coating model, the difference in the effects of the interaction of electromagnetic radiation with conductive layers located on a dielectric and metal substrate is demonstrated. It is shown that in order to achieve optimal (maximum) values of reflection and absorption of electromagnetic radiation in the appropriate frequency range, it is necessary to fit the appropriate layer thicknesses, specific conductivity, and permittivity.

The use of highly reflective screens may damage the electronic device itself. The reflection of electromagnetic radiation from the screen is the result of a mismatch between the impedances of the two media. Thus, in order to achieve high shielding efficiency in various frequency ranges, new and improved shield designs are required that can provide impedance matching and effectively absorb electromagnetic radiation.

In the review, all polymer composites are classified according to the type of filler. The issues of the interaction of a polymer with conductive fillers, the influence of the concentration of fillers and their location inside the matrix, and the structure of the nanocomposite on the mechanisms of electromagnetic interaction are considered. Current research on the application of polymer-based multilayer coatings for effective shielding of electromagnetic radiation is also discussed.

Porous materials, including synthetic foams, as well as layered and segregated structures with various fillers dispersed in the matrix have also shown their effectiveness in the production of shielding materials with a high level of electromagnetic interference. This is due to the appearance of multiple reflection and absorption due to their structure that leads to an increase in shielding efficiency.

Currently, there is no general procedure for shielding coatings production that can provide complete shielding without compromising other physical parameters. However, based on the developed polymer nanocomposites with different electro-physical parameters, electronic equipment designers have the opportunity to find solutions that meet the technical requirements for shielding from external electromagnetic interference.

Thus, the development of innovative polymer composite materials for shielding electronic devices from electromagnetic interference and excessive electromagnetic background is an important task that ensures the safe and uninterrupted operation of modern digital electronics and numerous other applications.

## Figures and Tables

**Table 1:** Results of measurement of *ρ_v_* composite materials based on *Fe*- and *Co*-containing nanoparticles.

Sample	*d_av_* (nm)	*C_met_* (wt. %)	*ρ_v_* (Ohm × m) at *U_app_*		
			10 V	100 V	1000 V
Polyethylene	-	0	-	4.9 × 10^14^	3.9 × 10^14^
Sample *Fe*-01 from *Fe(CO)_5_*	4.9	5	1.4 × 10^14^	1.2 × 10^14^	0.9 × 10^14^
Sample *Fe*-02 from *Fe(CO)_5_*	5.1	10	5.5 × 10^13^	5.5 × 10^13^	4.25 × 10^13^
Sample *Fe*-03 from *Fe(HCOO)_3_*	11.5	20	4.5 × 10^12^	3.0 × 10^12^	1.0 × 10^11^
Sample *Fe*-04 from *FeC_2_O_4_*	2.4	20	9.5 × 10^13^	5.1 × 10^13^	1.5 × 10^13^
Sample *Co*-01 from *Co(CH_3_COO)_2_*	8.3	20	2.8 × 10^13^	1.6 × 10^13^	5.5 × 10^12^

**Table 2:** Results of measurement of *ρ_v_* composite materials based on *Pb*-containing nanoparticles (author’s results).

*C_Pb_* (wt. %)	0	1	5	10
*ρ_v_* (Ohm × m)	0.83 × 10^14^	2.31 × 10^14^	1.05 × 10^14^	1.75 × 10^14^

**Table 3:** Measurement results for composite nanomaterials.

Sample	*C_met_* (wt. %)	*ε* (*f_op_* = 1 kHz)	*ε* (*f_op_* = 1 MHz)	*d_av_* (nm)
Polyethylene	0	2.36	2.25	-
Sample *Fe*-01 from *Fe(CO)_5_*	5	2.98	2.69	4.9
Sample *Fe*-02 from *Fe(CO)_5_*	10	3.63	3.32	5.1
Sample *Fe*-03 from *Fe(HCOO)_3_*	20	4.52	3.56	11.5
Sample *Fe*-04 from *FeC_2_O_4_*	20	3.67	3.37	2.4
Sample *Co*-01 from *Co(CH_3_COO)_2_*	20	3.96	3.01	8.3
Sample *Co*-02 from *Co(HCOO)_2_*	30	8.84	4.9	4.0
Sample *Co*-03 from *Co(HCOO)_2_*	30	18.9	17.7	4.0

**Table 4:** Results of measurements of *ε* Pb-containing composites at frequencies of 1 kHz and 1 MHz.

*C_Pb_* (wt. %)	0	1	5	10
*ε* (*f_op_* = 1 kHz)	2.36	2.21	2.29	2.40
*ε* (*f_op_* = 1 MHz)	2.25	2.12	2.23	2.32

**Table 5:** Results of measurements of *ε* of rhenium-containing composites at frequencies of 1 kHz and 1 MHz.

	HPPE	*Re*-01	*Re*-02	*Re*-03	*Re*-04	*Re*-05
*ε* (*f_op_* = 1 kHz)	2.36	2.77	2.35	2.49	2.68	2.44
*ε* (*f_op_* = 1 MHz)	2.25	2.68	2.24	2.35	2.39	2.37

**Table 6:** Characteristics of measurement ranges.

#	Operating Frequency (GHz)	Wavelength (mm)	Waveguide Cross Section (mm^2^)
1	17.44–25.95	11.5–17.2	11 × 5.5
2	25.95–37.5	8.0–11.5	7.2 × 3.4
3	37.5–53.57	5.6–8.0	5.2 × 2.6

**Table 7:** The value of residual magnetization (*M_r_*) and magnetization in a field with a value of 4.5 kOe (M_H_) and coercive force (*H_C_*) at room temperature and 100 °C for a sample that has been heat treated at different temperatures [173].

	*H_C_*	*M_r_*	*M_H_* (*H* = 4.5kOe)	*H_C_* (100 °C)
	(Oe)	(µ_B_)	(µ_B_)	(Oe)
The original sample	950	0.25	0.72	566
195 °C	720	0.16	0.48	
215 °C	570	0.14	0.50	
240 °C	490	0.15	0.56	
260 °C	270	0.10	0.55	230
290 °C	170	0.08	0.64	

**Table 8:** Values of residual magnetization (*M_R_*), magnetization in the field of 4.5 kOe (*M_H_*_=4.5_), and coercive force (*H_C_*) at room temperature and at 100 °C for the initial and calcined samples.

	*H_C_*	*M_R_*	*M_H_*_=4.5_	*H_C_* (100 °C)
	(Oe)	(µ_B_)	(µ_B_)	(Oe)
The original sample	590	0.35	1.05	590
Sample heated at 280 °C	590	0.62	1.96	590

**Table 9:** Magnetic susceptibility of test samples.

Sample	$\chi_{sp}$ (Experiment), 10^−9^ (m^3^/kg)	$\chi_{sp}$ [200], 10^−9^ (m^3^/kg)
*Al*	0.65 ± 0.02	0.61
*Zn*	−0.135 ± 0.005	−0.175 (298 K); $-0.190\;(\chi_{⇑}$, 293 K); $-0.145\;(\chi_{⊥}$, 293 K);
*Ho_2_Ti_2_O_7_*	157 ± 1	154

**Table 10:** Magnetic susceptibility of samples with $\chi_{sp}$ < 0.

Sample	$\chi_{sp}$ (Experiment), 10^−9^ (m^3^/kg)
HPPE	−0.75 ± 0.09
HPPE + 20% *Mo*	−0.44 ± 0.06
HPPE + 20% *Bi*	−0.72 ± 0.05
HPPE + 5% *Hg*	−0.86 ± 0.15
HPPE + 5% *Re*	−0.79 ± 0.07

**Table 11:** Parameters of linear regression and magnetic susceptibility of the samples with *M_S-0_* ≠ 0.

Sample	A (emu/g)	C (cm^3^/g)	χ_sp_ (Experiment), 10^−9^ (m^3^/kg)
HPPE + 10% *Cu*	(12.0 ± 0.7) × 10^−2^	(−8.2 ± 1.4) × 10^−3^	−0.65 ± 0.11
HPPE + 10% *Pb*	(4.9 ± 1.2) × 10^−2^	(−9.9 ± 2.4) × 10^−3^	−0.79 ± 0.19
HPPE + 40% *CeO_2_*	(1.90 ± 0.52) × 10^−2^	(−4.37 ± 0.95) × 10^−3^	−0.35 ± 0.08
HPPE + 20% *CeO_2_*	(1.54 ± 0.29) × 10^−2^	(−6.68 ± 0.61) × 10^−3^	−0.53 ± 0.05
HPPE + 40% *CdS*	(1.42 ± 0.18) × 10^−2^	(7.77 ± 0.36) × 10^−3^	−0.62 ± 0.03

**Table 12:** The coefficients of attenuation A, reflection R, and loss L of samples based on iron-containing nanoparticles synthesized from Fe(HCOO)_3_.

Sample	*C_Fe_* (wt. %)	*h* (mm)	*R*		*L*		*A* (dB)	
			25 GHz	30 GHz	25 GHz	30 GHz	25 GHz	30 GHz
*Fe*-05	30	2.9	0.61	0.53	0.32	0.42	11.60	13.0
*Fe*-03	20	0.95	0.35	0.22	0.0	0.09	1.35	1.6
PE	0	1.2	-	0.12	-	0.0	-	0.3

**Table 13:** The coefficients of attenuation *A*, reflection *R*, and loss *L* of samples of composite materials based on cobalt-containing nanoparticles.

Sample	*C_Fe_* (wt. %)	*h* (mm)	*R*	*L*	*A* (dB)
			30 GHz	30 GHz	30 GHz
*Co*-01	20	1.62	0.030	0.645	4.9
*Co*-02	30	2.11	0.085	0.575	4.7
*Co*-03	30	2.07	0.080	0.895	16.0
PE	0	1.2	0.120	0.0	0.3

**Table 14:** Values of attenuation and reflection coefficients for molybdenum-containing nanocomposites at a frequency of 30 GHz.

Sample	PE	*Mo* 7% in PE	*Mo* 20% in PE
*A* (dB)	0.3	0.8	1.0
R* (dB) (%)	–9.0 (12.5)	–8.5 (13.8)	–7.5 (17.5)
*h* (mm)	1.2	1.65	1.29

**Table 15:** The values of specific radio absorption *a* and reflection *R* coefficients in the microwave range for lead-containing composites at a frequency of 30 GHz.

*C_Pb_* (wt. %)	0	1	5	10
*a* (dB/cm)	2.0	0.3	1.3	0.9
*R*	0.08	0.12	0.12	0.14

**Table 16:** Values of the specific radio absorption and reflection coefficients in the microwave range for rhenium-containing composites at a frequency of 30 GHz.

	PE	*Re*-01	*Re*-02	*Re*-03	*Re*-04	*Re*-05
*a*, (dB/cm)	2.2	6.1	2.2	3.4	4.7	2.9
*R*	0.14	0.15	0.14	0.16	0.14	0.17

**Table 17:** Attenuation, reflection, and loss coefficients in the microwave range for composites on metal-containing nanoparticles and HPPE matrix.

Sample	*C_Met_* (wt. %)	*h* (mm)	*R*		*L*		*A* (dB)	
			25 (GHz)	30 (GHz)	25 (GHz)	30 (GHz)	25 (GHz)	30 (GHz)
PE	0	1.2	-	0.12	-	0.0	-	0.3
*Fe*-05	30	2.9	0.61	0.53	0.32	0.42	11.60	13.0
*Fe*-03	30	0.95	0.35	0.22	0.0	0.09	1.35	1.6
*Co*-01	20	1.62	-	0.03	-	0.65	-	4.9
*Co*-02	30	2.11	-	0.09	-	0.58	-	4.7
*Co*-03	30	2.07	-	0.08	-	0.90	-	16.0
*Mo*-04	7	1.65	-	0.14	-	0.03	-	0.8
*Mo*-08	20	1.29	-	0.18	-	0.03	-	1.0
*Pb*-01	1	1.93	-	0.12	-	0.0	-	0.3
*Pb*-04	10	1.93	-	0.14	-	0.05	-	0.9
*NiFe_2_O_4_*	10	1.01	-	0.13	-	0.06	-	0.9
*NiFe_2_O_4_*	20	1.03	-	0.15	-	0.07	-	1.1
*NiFe_2_O_4_*	30	1.17	-	0.22	-	0.12	-	1.8
*Re*-01	5	1.32	-	0.15	-	0.02	-	0.8
*Re*-02	5	1.36	-	0.14	-	0.0	-	0.3
*Re*-03	5	1.74	-	0.16	-	0.0	-	0.6
*Re*-04	5	1.71	-	0.17	-	0.0	-	0.8

**Table 18:** Electrophysical properties of composites based on PVC-filled plastics.

Filler	*d* (mm)	*ρ_v_* (Ohm × m)	*ε* (tan*δ*)	*R*	*Q*	Conditional Sample #
-	1.400	1.3 × 10^8^	4 (0.1)	0.13	0.02	01
	2.285	1.6 × 10^8^	4 (0.2)	0.10	0.01	02
	3.520	1.8 × 10^8^	4 (0.2)	0.03	0.04	03
1 mass. % TEG	1.645	0.6 × 10^8^	6 (0.1)	0.22	0.23	1TEG-01
	2.755	0.8 × 10^8^	6 (0.1)	0.25	0.43	1 TEG-02
	4.100	1.0 × 10^8^	6 (0.1)	0.13	0.51	1 TEG-03
2 mass. % TEG	1.922	0.8 × 10^8^	6 (0.1)	0.10	0.46	2 TEG-01
	2.802	0.7 × 10^8^	7 (0.1)	0.40	0.42	2 TEG-02
	4.287	0.8 × 10^8^	8 (0.1)	0.25	0.62	2 TEG-03
3 mass. % TEG	2.115	0.5 × 10^8^	13 (0.2)	0.46	0.39	3 TEG-01
	2.802	0.8 × 10^8^	17 (0.5)	0.27	0.57	3 TEG-02
	4.252	0.4 × 10^8^	19 (0.6)	0.30	0.63	3 TEG-03
*BaTiO_3_*	1.590	1.6 × 10^8^	7 (0.1)	0.25	0.11	BT-01
	2.407	1.4 × 10^8^	7 (0.1)	0.15	0.27	BT-02
	3.780	1.5 × 10^8^	7 (0.1)	0.09	0.32	BT-03
*BaTiO_3_* + CG	1.690	1.0 × 10^8^	10 (0.1)	0.08	0.44	BTCG-01
	2.785	0.8 × 10^8^	9 (0.1)	0.35	0.39	BTCG-02
*BaTiO_3_* + AMAG	1.655	4.0 × 10^9^	10 (0.1)	0.51	0.42	BTAM

**Table 19:** Three-layer combinations of composites based on PVC-filled plastics.

Layer 1		Layer 2		Layer 3	
# Sample	*h_1_* (mm)	# Sample	*h_2_* (mm)	# Sample	*h_Δ_* (mm)
2TEG-02	2.802	3TEG-02	2.802	01	1.400
2TEG-02	2.802	3TEG-02	2.802	BT-01	1.590
2TEG-02	2.802	3TEG-02	2.802	BTCG-01	1.690
2TEG-02	2.802	3TEG-02	2.802	BTAM	1.655
1TEG-02	2.755	3TEG-02	2.802	01	1.400
1TEG-02	2.755	3TEG-02	2.802	BT-01	1.590
1TEG-02	2.755	3TEG-02	2.802	BTCG-01	1.690
1TEG-02	2.755	3TEG-02	2.802	BTAM	1.655
1TEG-02	2.755	2TEG-02	2.802	01	1.400
1TEG-02	2.755	2TEG-02	2.802	BT-01	1.590
1TEG-02	2.755	2TEG-02	2.802	BTCG-01	1.690
1TEG-02	2.755	2TEG-02	2.802	BTAM	1.655

## Data Availability

Non applicable.

## Abbreviations

AMAG	amorphous magnetic alloy powder
CG	colloidal graphite
CGS	centimeter–gram–second system
CM	composite materials
CNT	carbon nanotubes
CVC	current-voltage characteristic
EMC	electromagnetic compatibility
EMF	electromagnetic field
EMR	electromagnetic radiation
EMW	electromagnetic waves
HPPE	high-pressure polyethylene
PVC	polyvinyl chloride
RAC	radio-absorbing coatings
RAM	radio-absorbing materials
RFS	radio frequency spectrum
SWR	standing wave ratio
TEG	thermally expanded graphite
VSWC	voltage standing wave coefficient
